# Chloroplast genomic resources for phylogeny and DNA barcoding: a case study on *Fritillaria*

**DOI:** 10.1038/s41598-018-19591-9

**Published:** 2018-01-19

**Authors:** Yu Bi, Ming-fang Zhang, Jing Xue, Ran Dong, Yun-peng Du, Xiu-hai Zhang

**Affiliations:** 1 Beijing Agro-Biotechnology Research Center, Beijing Academy of Agriculture and Forestry Sciences, Beijing, 100097 P. R. China; 2 Beijing Key Laboratory of Agricultural Genetic Resources and Biotechnology, Beijing Engineering Technology Research Center of Functional Floriculture, Beijing, 100097 P. R. China; 3 Engineering Research Center of Mt. Changbai Ecological Resources Development, Changhcun Sci-Tech University, Changchun, 130600 Jilin Province P. R. China

## Abstract

The genus *Fritillaria* comprises approximately 130 perennial herbaceous species. In the Pharmacopoeia of the People’s Republic of China, the bulbs of 11 *Fritillaria* species are used in Chinese herbal medicines. However, the traditional methods of morphological classification cannot accurately identify closely related species of *Fritillaria*. Previous studies have attempted to identify these species with universal molecular markers, but insufficient phylogenetic signal was available. In this study, the complete chloroplast genomes of eight *Fritillaria* species were compared. The length of the eight *Fritillaria* chloroplast genomes ranges from 151,009 bp to 152,224 bp. A total of 136 SSR loci were identified, including 124 polymorphic SSR loci. For large repeat sequences, 108 repeat loci and four types of repeats were observed. Ten highly variable regions were identified as potential molecular markers. These SSRs, large repeat sequences and highly variable regions provide important information for the development of genetic markers and DNA fingerprints. Phylogenetic analyses showed that the topological structures of all data sets (except the IR regions) were in complete agreement and well resolved. Overall, this study provides comprehensive chloroplast genomic resources, which will be valuable for future studies of evolution and species identification in *Fritillaria*.

## Introduction

The genus *Fritillaria*, in the family Liliaceae, includes approximately 130 species of perennial herbaceous flowers^1,2^. It is distributed in the temperate regions of the northern hemisphere, mainly in Central Asia, the Mediterranean region and North America^3^. The genus *Fritillaria* is characterized by fleshy bulbs with farinaceous scales, petiolate basal leaves, sessile cauline leaves, bisexual and usually nodding flowers, perigone campanulate with segments erect in the distal part, and loculicidal capsules with seeds that are usually winged^2–4^. Members of the *Fritillaria*, as one of the most desired ornamental plants, have great agronomic and economic importance. *Fritillaria* bulbs are widely used population growth and commercial distribution. In addition, *Fritillaria* bulbs have been widely used as herbs in traditional Chinese medicine for more than 2000 years.

The *Fritillaria* plants used in herbal medicines are included in the Pharmacopoeia of the People’s Republic of China—*F. hupehensis* Hsiao & K.C.Hsia, *F. thunbergii* Miq., *F. walujewi* Regel, *F. pallidiflora* Schrenk, *F. usuriensis* Maxim., *F. przewalskii* Maxim., *F. unibracteata* Hsiao & K.C.Hsia, *F. delavayi* Franch., *F. cirrhosa* D.Don, *F. taipaiensis* P.Y.Li, and *F. unibracteata* Hsiao & K.C.Hsia var. *wabuensis* (S.Y.Tang & S.C.Yueh) Z.D.Liu, Shu Wang & S.C.Chen^5^. Within the realm of Chinese medicine, *Fritillaria* bulbs have heat clearing, expectorant, antitussive, detoxifying and analgesic properties and are used to treat conditions such as dry coughing, sputum with blood, umbilical carbuncle and acute mastitis^5–7^. Moreover, modern pharmacological studies have demonstrated that *Fritillaria* bulb extracts have therapeutic benefits for leukemia, liver cancer and cervical cancer as well as antitumor activity^6,8^. Each *Fritillaria* species used in traditional Chinese medicine has its own unique efficacy and active biological ingredients. Therefore, *Fritillaria* bulbs of both ornamental and medicinal must be accurately identified. Given the difficulty in morphological classification, more effective molecular markers are needed to identify *Fritillaria* species.

The genus *Fritillaria* is currently divided into eight subgenera: *Davidii*, *Liliorhiza*, *Japonica*, *Fritillaria*, *Rhinopetalum*, *Petilium*, *Theresia* and *Korolkowia*^9^. The nuclear DNA internal transcribed spacer (ITS) and several plastid genome regions (*trnL*-*trnF*, *matK* and *rpl16*) have frequently been used for phylogenetic analysis of *Fritillaria* species, but previous studies have found that these markers provided insufficient phylogenetic signal^10–15^. Ronsted *et al*.^10^ recovered strong support for two major clades in the genus *Fritillaria*, one comprising species from the mainly North American subgenus *Liliorhiza* and the other made up of species from the seven remaining subgenera. In addition, they constructed a phylogenetic tree based on *matK* and *rpl16* sequences, and that tree did not resolve *Fritillaria* as monophyletic. However, Day *et al*.^11^ suggested that two strongly supported clades were unresolved with respect to each other and to *Lilium* in the Bayesian tree with *matK* and *rbcL* sequences. In other studies, phylogenetic trees have either been based on small, geographically restricted samples of species or had low support for the relationships recovered^12,15^. In addition, Day *et al*.^11^ attempted to develop low-copy nuclear gene regions as markers, but none of the regions examined could be amplified from all taxa tested. Simultaneously, Li and Song *et al*.^16^ demonstrated that single molecule, real-time (SMRT) sequencing yields high-quality *Fritillaria* chloroplast genomes for sensitive SNP detection and comparative analyses. However, they calculated the variation within only the protein-coding genes and thus found that *rps19* had the greatest interspecific variation. However, previous comparison analyses of whole cp genomes have reported that the noncoding regions had higher proportions of variability. Therefore, the analysis based on the whole chloroplast genome sequence could provide more sufficient phylogenetic signals and identify more effective molecular markers.

Chloroplasts are essential organelles in photosynthetic algae and plant cells and play a crucial role in sustaining life^17,18^. Chloroplast genomes are mainly inherited from the maternal parent^19^. The cp genome typically has a double-stranded, circular molecular structure; a length of 120–220 kb; and 120–140 protein-coding genes^20,21^. The quadripartite structure of the chloroplast genome includes a large single copy (LSC) region, a small single copy (SSC) region, and two copies of an inverted repeat region (IRA and IRB). Due to the high conservation of chloroplast genomes compared to nuclear and mitochondrial genomes, partial chloroplast genome sequences have often been used for phylogenetic studies and species identification^10,12,22–24^. However, these incomplete sequences contain insufficient information to provide the high resolution necessary to differentiate closely related taxa, particularly some taxa below the species level with unclear taxonomic relationships. Complete chloroplast genome sequences are valuable for deciphering phylogenetic relationships between closely related taxa and for improving our understanding of the evolution of plant species. Wu *et al*.^25^ found that the *Oncidium* chloroplast genome provides useful molecular markers to resolve phylogenetic relationships among 15 commercial varieties within the Oncidiinae at the species level and to help determine their parental origins. Li and Li *et al*.^26^ developed chloroplast microsatellite markers in the cotton genus to reveal the diversity and differentiation of *Gossypium* species during evolution. Ma *et al*.^27^ constructed high-resolution phylogenetic trees with 25 complete cp genomes of bamboo species to resolve the deep-level relationships of Arundinarieae. Therefore, complete chloroplast genome sequences are valuable for deciphering the phylogenetic relationships among closely related taxa, for improving our understanding of the evolution of plant species, for exploiting DNA barcodes to identify varieties, and for promoting germplasm innovation.

In this study, we present four subgenera complete cp genomes of *Fritillaria* (*F. eduardii*, *F. karelinii*, *F. meleagroides*, *F. persica*) obtained through next-generation sequencing (NGS) and genomic comparative analyses with four previously published cp genome sequences of the subgenera *Fritillaria* downloaded from NCBI (National Center for Biotechnology Information, https://www.ncbi.nlm.nih.gov). Here, we identify simple sequence repeats (SSRs), larger repeat sequences and highly variable regions for developing DNA barcodes and test the feasibility of phylogenetic analyses using the chloroplast genome.

## Results and Discussion

### Genome sequencing and assembly

In the four *Fritillaria* species sequenced in this study, 3,629,318 to 56,287,190 paired-end raw reads were generated with an average read length of 150 bp on the Illumina Sequencing System. From 50,995 to 133,071 reads were extracted to assemble complete chloroplast genome sequences with 50.25×to 131.45×coverage. The four novel *Fritillaria* cp genome sequences were preserved in GenBank (Table 1). The four junction regions were validated by PCR-based sequencing in each of the four *Fritillaria* cp genomes. The four *Fritillaria* cp genome sizes ranged from 151,803 bp (*F. persica*) to 152,224 bp (*F. eduardii*; Table 1).

**Table 1:** Summary of the sequencing data for four *Fritillaria* species.

Species	Raw data no.	Mapped read no.	Mapped to reference genome (%)	cp gemome coverage (X)	cp gemome length (bp)	Accession number in Genbank
*F. eduardii*	7,101,661	50,995	0.72%	50.25	152,224	MF947708
*F. karelinii*	8,110,794	81,422	1.00%	80.29	152,118	KX354691
*F. meleagroides*	56,287,190	133,071	0.24%	131.45	151,846	MF947710
*F. persica*	3,629,318	60,458	1.67%	59.74	151,803	MF947709

### Comparative analysis of *Fritillaria* chloroplast genomes

**Figure 1:**
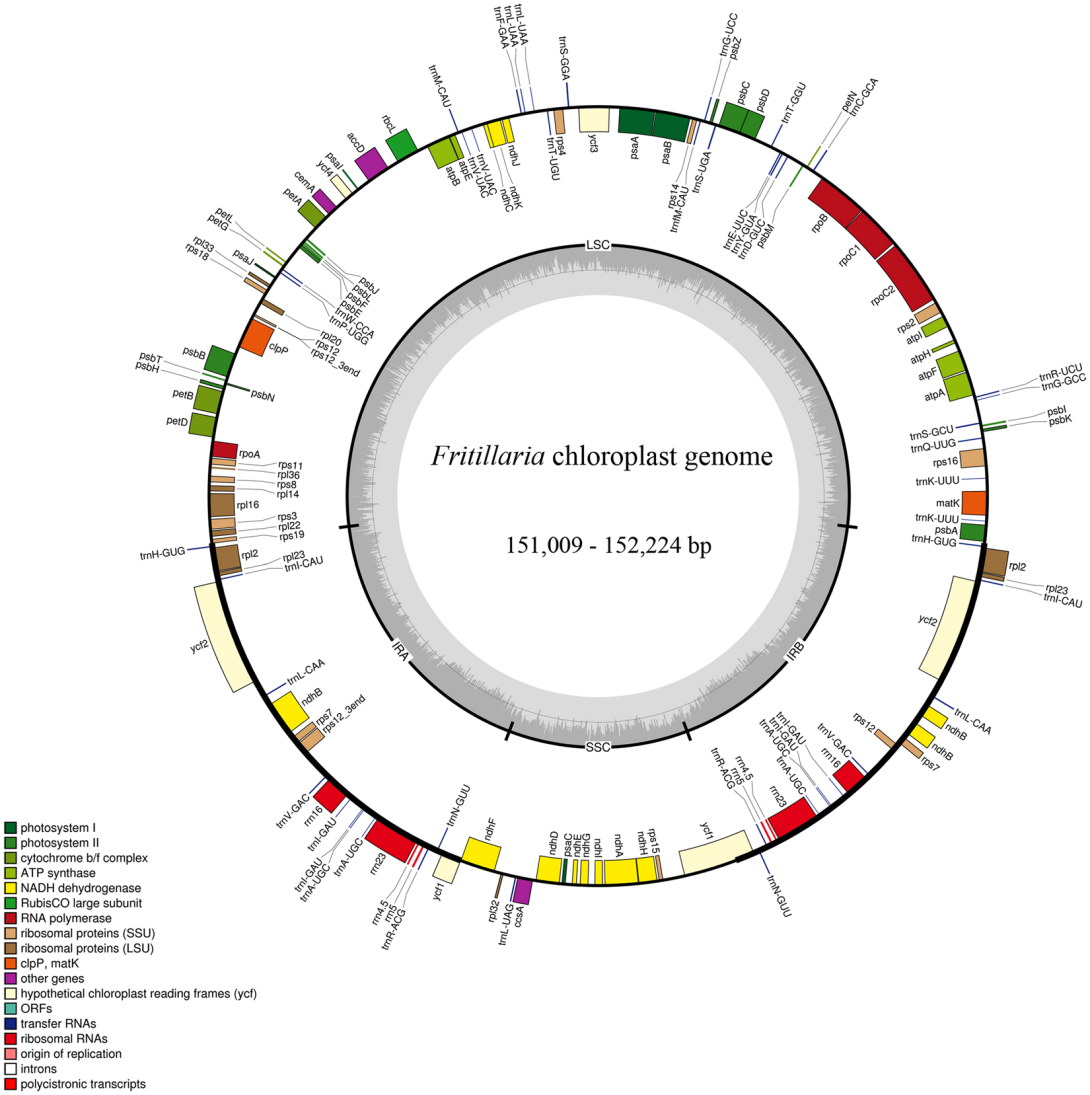
Gene map of the *Fritillaria* chloroplast genome. The genes inside and outside of the circle are transcribed in the clockwise and counterclockwise directions, respectively. Genes belonging to different functional groups are shown in different colors. The thick lines indicate the extent of the inverted repeats (IRa and IRb) that separate the genomes into small single copy (SSC) and large single copy (LSC) regions.

The eight *Fritillaria* cp genomes ranged from 151,009 bp (*F. unibracteata* var. *wabuensis*) to 152,224 bp (*F. eduardii*; Fig. 1, Table 2). The chloroplast genomes consisted of circular double-stranded DNA and displayed a quadripartite structure, including an LSC region of 81,286 bp-82,130 bp, an SSC region of 16,962 bp-17,949 bp, and a part of the IR regions of 25,887 bp-26,387 bp. The overall A + T content of the whole genomes was 63.00%-63.05% (Table 2). *Fritillaria* cp genomes have high A + T content, which has been widely observed in many sequences from angiosperm cp genomes^28–32^.

**Table 2:** Summary of complete chloroplast genomes of *Fritillaria* species.

Species	Total	LSC	IR	SSC	Total	Protein coding genes	tRNA	rRNA	AT%	Accession number in Genbank
*F. cirrhosa*	151,991	81,769	26,343	17,535	128	82	38	8	63.05%	KF769143
*F. eduardii*	152,224	81,991	26,353	17,527	128	82	38	8	63.01%	MF947708
*F. hupehensis*	152,145	81,894	26,349	17,553	128	82	38	8	63.03%	KF712486
*F. karelinii*	152,118	81,875	26,387	17,469	128	82	38	8	63.05%	KX354691
*F. meleagroides*	151,846	82,130	26,377	16,962	128	82	38	8	63.04%	MF947710
*F. persica*	151,803	81,634	26,330	17,509	128	82	38	8	63.00%	MF947709
*F. taipaiensis*	151,691	81,437	26,352	17,550	128	82	38	8	63.03%	KF769144
*F. unibracteata* var. *wabuensis*	151,009	81,286	25,887	17,949	128	82	38	8	63.03%	KF769142

A total of 128 coding genes were identically annotated in the same order, consisting of 82 protein-coding genes, 38 tRNA genes, and 8 rRNA genes (Fig. 1, Tables 2 & S4). Nineteen duplicated genes were found in the IR regions, as well as 7 protein-coding genes, 8 tRNA genes and 4 rRNA genes. Twenty-two protein-coding genes and 5 tRNA genes contained introns; 25 genes had a single intron, whereas 2 genes had two introns (Table S4). These results showed that the gene number and gene order, and thus the cp genome structure, of the genus *Fritillaria* were highly conserved, and similar effects have also been found in other genera, such as *Lilium*^30,32^, *Epimedium*^29^, *Rehmannia*^31^ and *Lagerstroemia*^33^.

**Figure 2:**
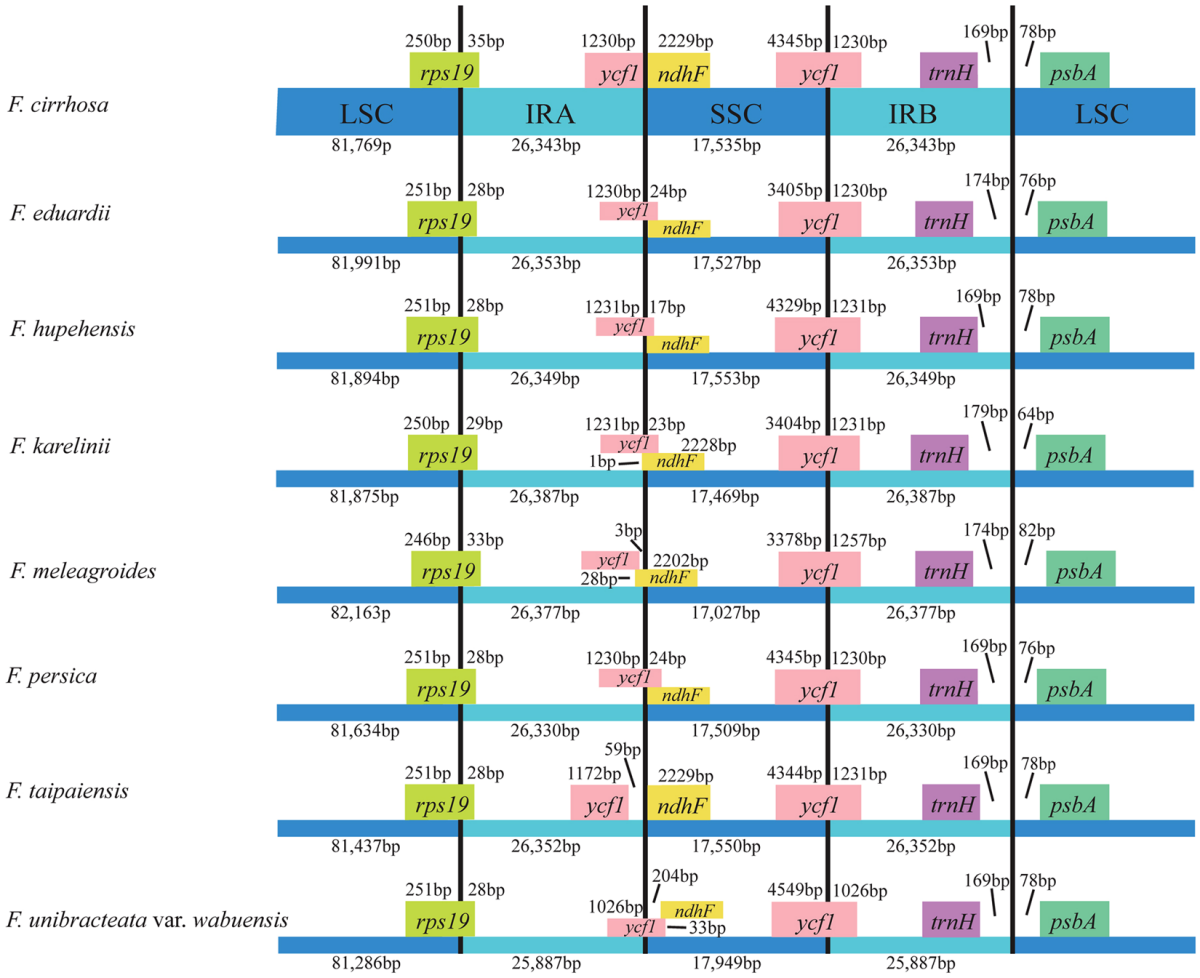
Comparisons of LSC, SSC, and IR region borders among the eight *Fritillaria* chloroplast genomes.

Although the IR regions are highly conserved, the expansion and contraction of IR region boundaries are considered to be the main mechanisms of length variation in the cp genomes of angiosperms^20,34,35^. In the four boundary regions (LSC, IRA, SSC, IRB) of eight *Fritillaria* cp genomes, the gene *rps19* crossed the LSC/IRA boundary, the gene *ycf1* crossed the SSC/IRB boundary, and the IRB/LSC border was in the intergenic region *trnH-psbA*. However, the IRA/SSC boundary in *Fritillaria* cp genomes contained some obvious differences (Fig. 2). In *F. eduardii*, *F. hupehensis*, *F. persica* and *F. unibracteata* var. *wabuensis*, the gene *ycf1* extended 17 bp-204 bp into the SSC region, which also had a 17 bp-33 bp overlap with *ndhF*. In *F. meleagroides*, the IRA/SSC border was positioned in the *ndhF* gene, which had 2225 bp in the SSC region and 28 bp in the IRA region, and *ndhF* also had a 25 bp overlap with *ycf1* in the IRA region. Moreover, both *ycf1* and *ndhF* crossed the IRA/SSC region in *F. karelinii*, *ycf1* with 23 bp located in the SSC region and *ndhF* with 1 bp in the IRA region. In *F. taipaiensis, ndhF* was in the IRA region, while *ycf1* and 59 noncoding bases were in the SSC region. In *F. cirrhosa*, the IRA/SSC border did not intersect any genes; this border was located at the junction between *ndhF* and *ycf1*. In *Fritillaria* cp genomes, variations in the IR and SSC border regions resulted in the length variation of these four regions and the whole cp genome sequences.

Illuminating the whole chloroplast genome of *Fritillaria* and the nucleotide diversity of the four fundamental regions (LSC, SSC, IRs), these results verify that the *Fritillaria* chloroplast genome is highly conserved, and the whole chloroplast genome has 2744 variable sites. The LSC and SSC regions contribute 1919 and 569 variable sites, respectively. The number of parsimony-informative sites is 545 and 191, respectively. In contrast, the IR regions have the lowest interspecific variation, with only 121 variable sites and 27 parsimony-informative sites (Table 3). Similarly, the value of nucleotide diversity in the IR regions is the lowest (0.00142). The level of sequence divergence in the SSC region is the highest (0.01122; Table 3). This study shows that the IR regions are the most conserved, in accordance with results from the chloroplast genomes of many plants reported previously^30,33^. However, some studies have reported that the LSC region had higher divergence than the SSC and IR regions^16^.

**Table 3:** Variable site analyses in *Fritillaria* chloroplast genomes.

	Number of sites	Number of variable sites	Number of parsimony informative sites	Nucleotide Diversity
LSC	84,180	1919	545	0.00770
SSC	18,289	569	191	0.01122
IR	26,486	121	27	0.00142
Complete cp genome	154,988	2744	796	0.00592

The differences and evolutionary divergences among eight *Fritillaria* cp genomes were compared using nucleotide substitutions and sequence distance. Across all eight species, the p-distance is 0.0016–0.0084, and the value of nucleotide differences is 243–1234. In subgenus *Fritillaria*, the p-distance is between 0.0016–0.0026 among the five species *F. cirrhosa*, *F. hupehensis*, *F. taipaiensis* and *F. unibracteata* var. *wabuensis*, while the p-distance between *F. meleagroides* and those five species is 0.0077–0.0082 (Table 4). One possible reason for this result is that subgenus *Fritillaria* is probably a polyphyletic group, rather than a monophyletic group^9,11^.

**Table 4:** Numbers of nucleotide substitutions and sequence distance in eight complete cp genomes. The upper triangle shows the number of nucleotide substitutions and the lower triangle indicates the number of sequence distance in complete cp genomes.

	*F. cirrhosa*	*F. eduardii*	*F. hupehensis*	*F. karelinii*	*F. meleagroides*	*F. persica*	*F. taipaiensis*	*F. unibracteata* var.* wabuensis*
*F. cirrhosa*		0.0053	0.0025	0.0082	0.0081	0.0056	0.0019	0.0019
*F. eduardii*	787		0.0050	0.0082	0.0083	0.0042	0.0054	0.0054
*F. hupehensis*	373	737		0.0079	0.0077	0.0052	0.0026	0.0026
*F. karelinii*	1215	1217	1165		0.0071	0.0083	0.0083	0.0083
*F. meleagroides*	1200	1218	1141	1052		0.0084	0.0082	0.0082
*F. persica*	827	626	771	1221	1234		0.0057	0.0056
*F. taipaiensis*	283	799	380	1230	1205	836		0.0016
*F. unibracteata* var. *wabuensis*	277	793	391	1221	1208	825	243	

### SSRs and large repeat sequences

An SSR is a repetitive unit consisting of 1–6 nucleotides, which is also called a microsatellite or short tandem repeat (STR)^36^. These repetitive units show codominant inheritance, high repeatability, and high variability in heterozygotes and are thus effective molecular genetic markers in plant population genetics, evolution, species identification and ecology^37–42^. In this study, numerous SSR loci were found through the comparative analysis of *Fritillaria* chloroplast genome sequences. In total, five types of SSR (mononucleotide, dinucleotide, trinucleotide, tetranucleotide and pentanucleotide repeats) were detected based on the comparison of eight *Fritillaria* cp genomes. There were no hexanucleotide repeats. Each *Fritillaria* cp genome had 60–85 SSRs, and a total of 576 SSRs was present in the *Fritillaria* cp genomes altogether. The lengths of these SSRs ranged from 10 to 24 bp (Tables 5, S5 & S6). There were 136 SSR loci in the aligned *Fritillaria* chloroplast genome, including 124 polymorphic SSRs. The most abundant type of SSR was mononucleotide repeats (393 repeats in 89 loci), followed by dinucleotide repeats (103 repeats in 24 loci), tetranucleotide repeats (62 repeats in 12 loci), trinucleotide repeats (14 repeats in 6 loci), and pentanucleotide repeats (4 repeats in 5 loci) (Tables 5 & S6). Mononucleotide repeats were also found to be the most abundant in *Lilium*, *Lagerstroemia* and *Epimedium*^29,30,33^. Thus, mononucleotide repeats may contribute more to heritable variation than the other kinds of SSR do. In this study, all mononucleotides were composed of A/T, which was similar to previous results in *Cardiocrinum*^28^ and *Lilium*^30^.

**Table 5:** Simple sequence repeats (SSRs) in the eight *Fritillaria* cp genomes.

Species	SSR loci no.	PolyM. loci no.	PolyM. loci (100%)	mono-	di-	tri-	tetra-	penta-	Location			Region		
									IGS	Intron	CDS	LSC	IR	SSC
*F. cirrhosa*	74	62	83.78%	53	13	2	6	0	46	13	15	57	4	13
*F. eduardii*	60	48	80.00%	39	12	1	8	0	35	11	14	42	4	14
*F. hupehensis*	85	73	85.88%	58	15	2	8	2	53	18	14	63	6	16
*F. karelinii*	61	49	80.33%	40	11	1	9	0	34	14	13	44	4	13
*F. meleagroides*	82	70	85.37%	55	16	2	8	1	50	17	15	63	6	13
*F. persica*	62	50	80.65%	40	10	3	9	0	34	12	16	42	4	16
*F. taipaiensis*	78	66	84.62%	54	14	2	7	1	47	17	14	58	4	16
*F. unibracteata* var. *wabuensis*	74	62	83.78%	54	12	1	7	0	45	15	14	55	4	15
Total Loci	136	124	91.18%	89	24	6	12	5	92	24	20	103	6	27

These 136 SSR loci were mainly located in the LSC region (103 SSRs), followed by the SSC region (24 SSRs) and a minority in the IR regions (6 SSRs; Tables 5 & S5). Only one SSR locus crossed the IRA/SSC border; this locus was located in the protein-coding gene *ycf1* in the cp genome of *F. unibracteata* var. *wabuensis*. Moreover, SSRs in the *Fritillaria* cp genome were distributed mainly in the intergenic spacers (92 SSRs), with others dispersed at similar levels in the introns (24 SSRs) and coding DNA sequences (CDS; 20 SSRs). The SSR loci in the CDS regions were located in nine protein-coding genes (*matK*, *rpoC1*, *rpoC2*, *cemA*, *ndhD*, *ndhG*, *ndhH*, *ycf2*, and *ycf1*) of the *Fritillaria* cp genome (Table S5). Lu *et al*.^28^ observed that 15 different SSRs were located in eight protein-coding genes (*ycf1*, *cemA*, *rpoC2*, *ycf2*, *ndhH*, *rpl22*, *ndhD*, and *ndhE*) of three *Cardiocrinum* chloroplast genomes. Xu *et al*.^33^ found that 63 SSRs were located in eight CDS regions (*rpoA*, *rpoB*, *rpoC2*, *cemA*, *ndhD*, *ndhF*, *ycf1*, and *ycf2*) of *Lagerstroemia* cp genomes. Therefore, strong evidence indicates that SSR loci can be used for species identification and phylogenic study when the SSRs in plant chloroplast genomes show abundant variation.

**Figure 3:**
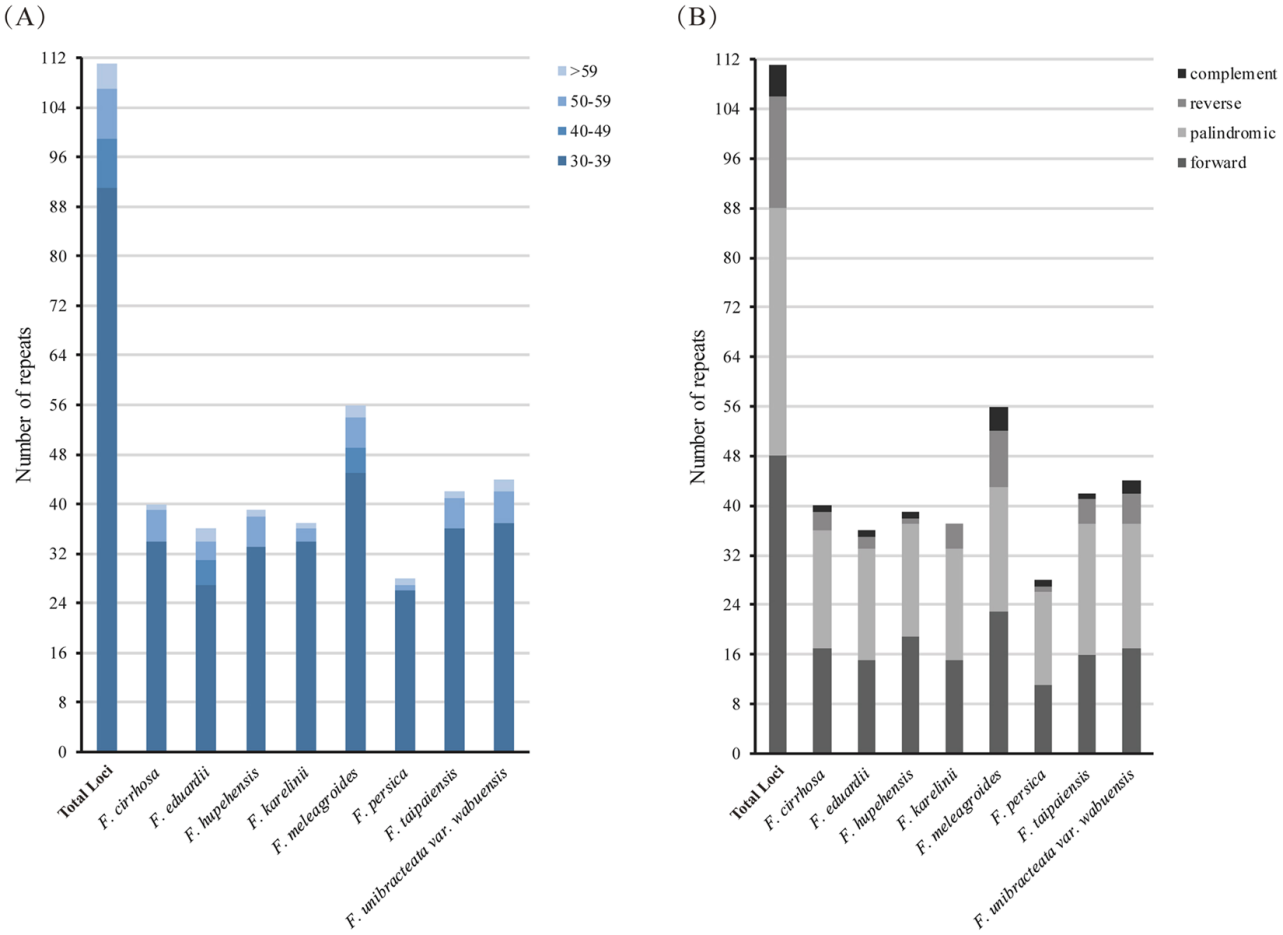
Analysis of large repeat sequences in the eight *Fritillaria* chloroplast genomes. (**A**) Frequency of repeats by length; (**B**) Frequency of repeat type.

In most angiosperm plants, frequent variation in repeat regions occurs due to illegitimate recombination and slipped-strand mispairing and plays an important role in sequence rearrangement and variation in cp genomes^29,43^. The large repeat sequences of the eight *Fritillaria* cp genomes were analyzed using REPuter, and 319 repeats (at least 30 bp long per repeat unit with hamming distance = 3), including forward (direct), reverse, complement and palindromic (inverted) repeats, were found (Fig. 3, Tables 6 & S7). The number of large repeat sequences ranged from 28 to 56 in each *Fritillaria* cp genome. Repetitions with the same lengths in the same region was regarded as repeat loci. A total of 108 repeat loci were found in the eight *Fritillaria* cp genomes, including 47 forward repeats, 38 palindromic repeats, 18 reverse repeats and 5 complement repeats. Overall, the lengths of the repeats ranged from 30 to 203 bp, and copy lengths with 30–39 bp (88 repeat loci) were the most common, while those with more than 59 bp (4 repeat loci) were the least common. In addition, 15 repeat loci were shared among the eight *Fritillaria* cp genomes. Moreover, *F. cirrhosa*, *F. eduardii*, *F. hupehensis*, *F. karelinii*, *F. meleagroides*, *F. persica*, *F. taipaiensis* and *F. unibracteata* var. *wabuensis* had 2, 9, 4, 12, 24, 2, 3, and 6 unique repeats, respectively. In addition, the quantity of identified repeat sequences is sensitive to the parameters, namely hamming distance. When the hamming distance reduced from 3 to 0, that is to say that the stringency was increased, the number of repeat sequences was narrowed to 205, 120 and 54 respectively (Table 6). When sequence identity was equal to 100% (hamming distance is 0), only forward and palindromic repeats were found. Meanwhile, according to the statistics it was detected that palindromic repeats were the most, then complement repeats were the least. These large repeat loci may provide abundant information as genetic markers for further development in phylogenetic and genetic analyses of *Fritillaria* species.

**Table 6:** The large repeated sequences in the eight *Fritillaria* cp genomes with different hamming distance.

Species	Hamming Distance = 3					Hamming Distance = 2					Hamming Distance = 1					Hamming Distance = 0		
	F	R	C	P	all	F	R	C	P	all	F	R	C	P	all	F	P	all
*F. cirrhosa*	17	3	1	19	40	11	3	0	14	28	4	0	0	9	13	1	3	4
*F. eduardii*	15	2	1	18	36	11	1	0	15	27	9	0	0	11	20	5	6	11
*F. hupehensis*	19	1	1	18	39	12	1	0	13	26	6	0	0	9	15	0	4	4
*F. karelinii*	15	4	0	18	37	9	3	0	11	23	6	1	0	7	15	3	6	9
*F. meleagroides*	22	9	4	18	53	11	4	2	12	29	8	0	1	10	19	5	8	13
*F. persica*	11	1	1	15	28	5	1	0	10	16	3	0	0	7	10	0	4	5
*F. taipaiensis*	16	4	1	21	42	10	2	0	16	28	4	0	0	10	14	0	4	4
*F. unibracteata* var. *wabuensis*	17	5	2	20	44	10	3	0	15	28	4	0	0	10	14	0	4	4
Total repeated sequences	132	29	11	147	319	79	18	2	106	205	44	1	1	73	120	14	39	54

F: forward (direct) matching; R: reverse matching; C: complement matching; P: palindromic (inverted) matching.

### Identification of highly variable regions

**Figure 4:**
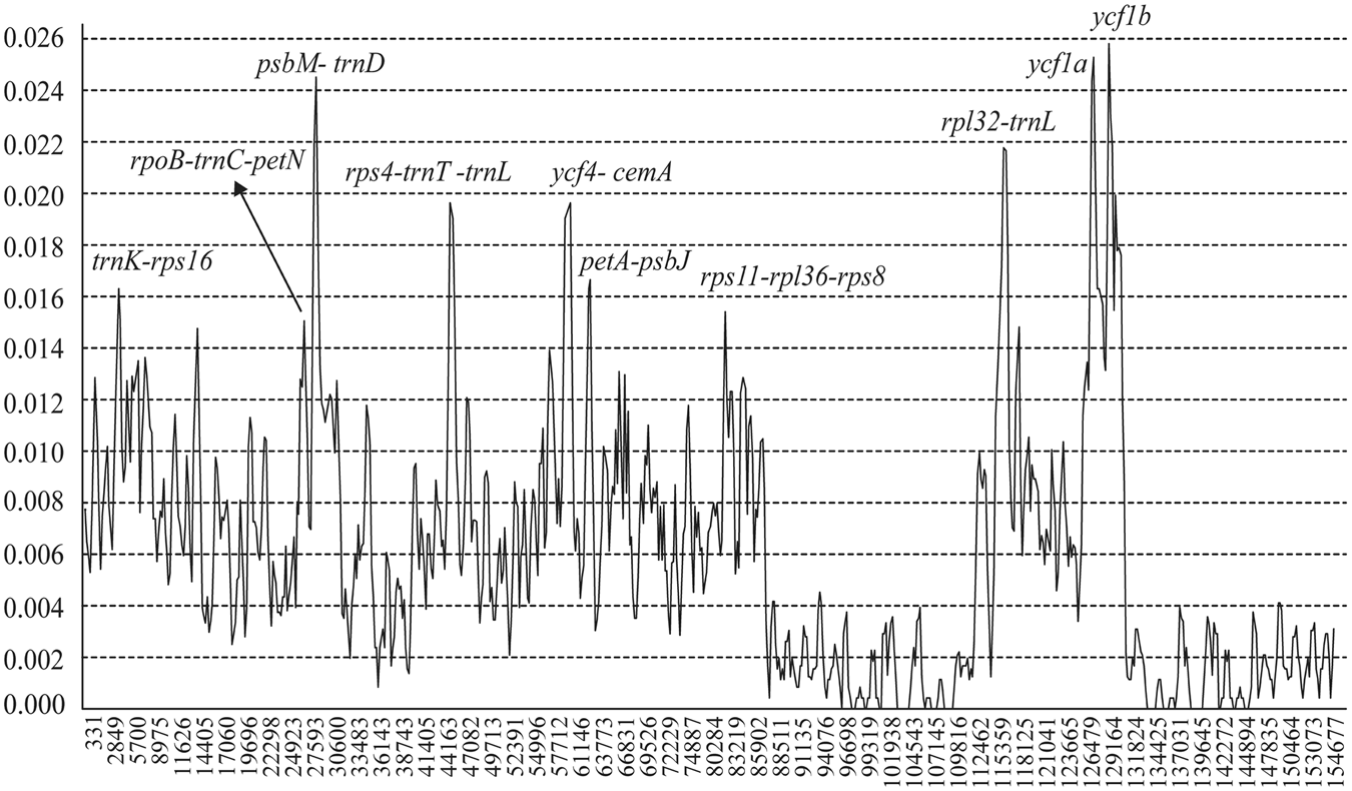
Sliding window analysis of the entire chloroplast genome of eight *Fritillaria* species (window length: 600 bp; step size: 200 bp). X-axis: position of the midpoint of a window; Y-axis: nucleotide diversity of each window.

Highly variable regions of chloroplast genomes can be used to identify closely related species more accurately and provide important information for phylogenetic study^30,44^. Nucleotide diversity was calculated with a sliding window (window length = 600 bp and step size = 200 bp) to estimate the divergence level of different regions in the *Fritillaria* cp genomes. The values of nucleotide diversity ranged from 0–0.02583 (Fig. 4). Ten regions with relatively high variability were selected as potential molecular markers for the study of species identification and phylogeny in *Fritillaria*. These 10 highly variable regions in the *Fritillaria* cp genome included eight intergenic spacer regions (*trnK*-*rps16*, *rpoB*-*trnC*-*petN*, *psbM*-*trnD*, *rps4*-*trnT*-*trnL*, *ycf4*-*cemA*, *petA*-*psbJ*, *rps11*-*rpl36*-*rps8*, and *rpl32*-*trnL*) and two protein-coding regions (*ycf1a* and *ycf1b*; Fig. 4, Table 7). Three regions (*ycf1a*, *ycf1b* and *rpl32*-*trnL*) were located in the SSC, and the other 7 regions were in the LSC region. All nucleotide diversity values in the IR regions were less than 0.005, and no highly divergent sequences were found, so those regions were considered to be highly conserved. The ten highly variable regions included 447 variable sites, including 142 parsimony-informative sites, and their nucleotide diversity values ranged from 0.01519–0.02571. The region of *ycf1a* showed the highest variability; the next most variable regions were *ycf1b*, *rpl32-trnL* and *psbM-trnD*; and the diversity level of *rpoB-trnC-petN* was the lowest. These selected sequences from highly variable regions provide a valuable resource for the study of species identification, breeding direction, phyletic evolution and population genetics.

**Table 7:** Ten regions of highly variable sequences of *Fritillaria*.

No.	High variable marker	Length	Variable sites	Parsimony informative sites	Nucleotide diversity
1	*trnK-rps16*	877	31	10	0.01557
2	*rpoB-trnC-petN*	661	30	8	0.01519
3	*psbM- trnD*	889	56	11	0.02260
4	*rps4-trnT -trnL*	965	42	11	0.01851
5	*ycf4- cemA*	1276	42	14	0.01786
6	*petA-psbJ*	809	43	8	0.01634
7	*rps11-rpl36-rps8*	582	29	8	0.01643
8	*rpl32-trnL*	816	48	17	0.02432
9	*ycf1a*	1010	62	26	0.02571
10	*ycf1b*	1001	64	29	0.02229
11	Combine	8886	447	142	0.01951

### Phylogenetic analyses

**Figure 5:**
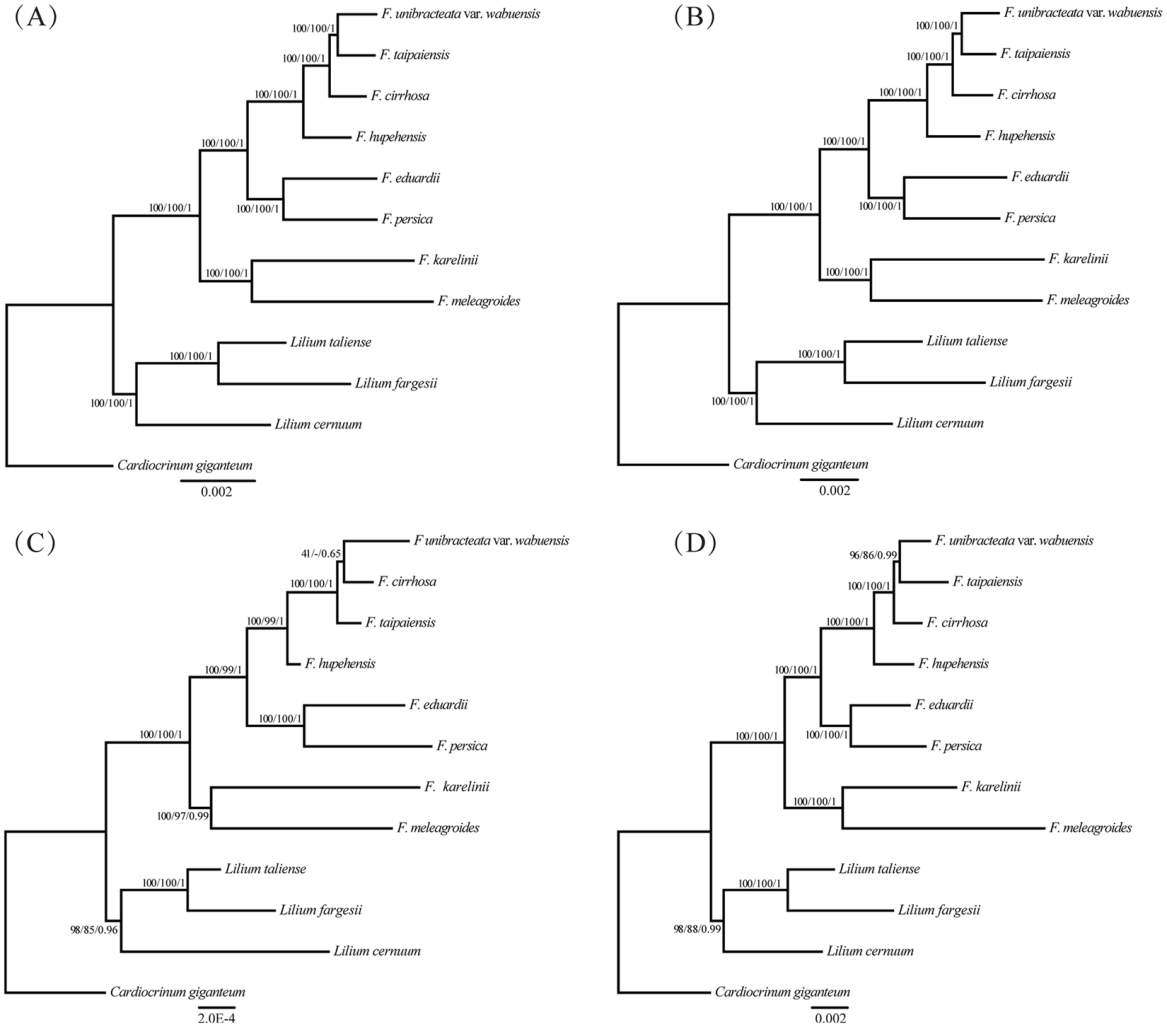
Phylogenetic relationships of the eight *Fritillaria* species inferred from maximum likelihood (ML), maximum parsimony (MP) and Bayesian (BI) analyses of different data partitions. (**A**) Whole chloroplast genome. (**B**) LSC region. (**C**) IR region. (**D**) SSC region. Numbers above nodes are support values with ML bootstrap values on the left, MP bootstrap values in the middle, and Bayesian posterior probabilities (PP) values on the right.

**Figure 6:**
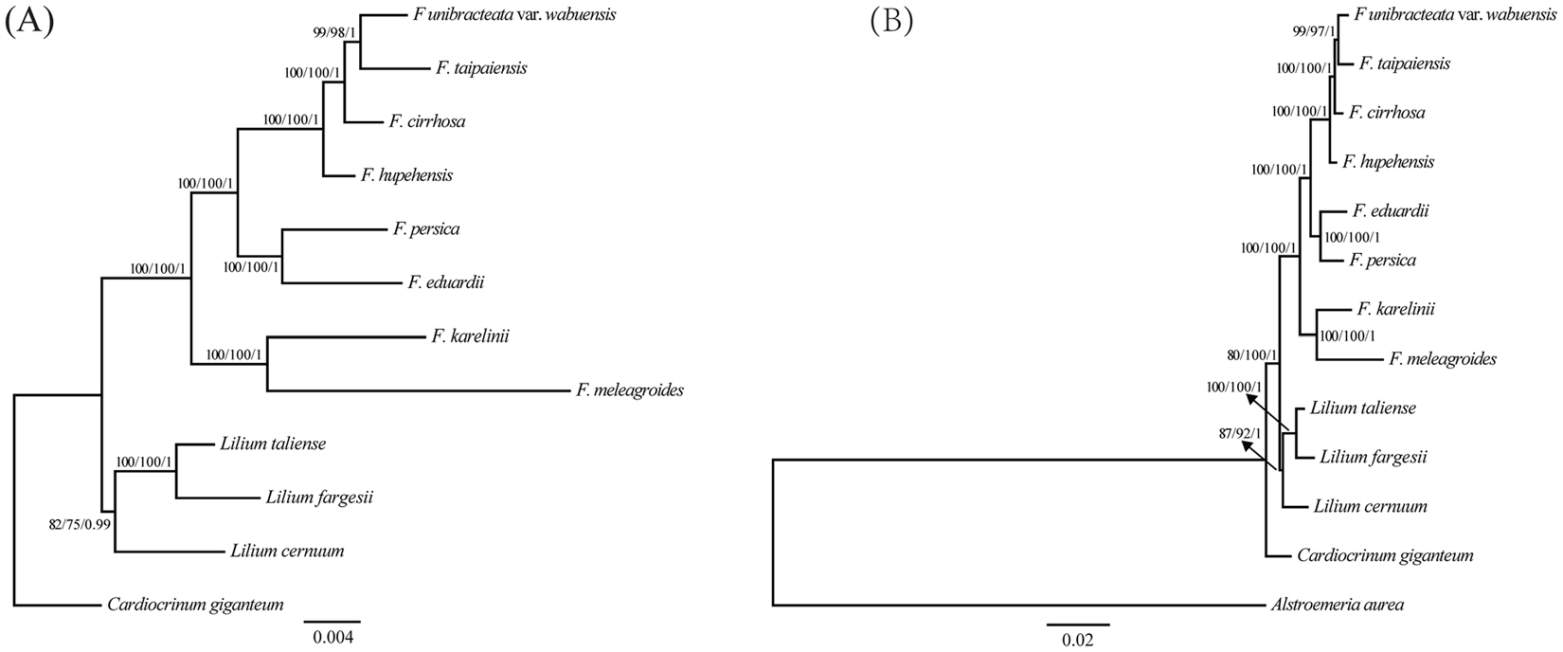
Phylogenetic relationships of the eight *Fritillaria* species inferred from maximum likelihood (ML), maximum parsimony (MP) and Bayesian (BI) analyses of highly variable regions. (**A**) *Cardiocrinum giganteum* as outgroup. (**B**) *Alstroemeria aurea* as outgroup. Numbers above nodes are support values with ML bootstrap values on the left, MP bootstrap values in the middle, and Bayesian posterior probabilities (PP) values on the right.

With the advent of NGS technology, some problems of angiosperm phylogeny and related species identification within categories and between species have been solved using chloroplast genome sequences for multiple groups, such as Myrtaceae^45^, Araceae^46^, Arundinarieae^27^, *Citrus*^47^, *Oncidium*^25^, and *Gossypium*^26^. In the present study, five data sets (complete chloroplast genomes, LSC regions, IR regions, SSC regions and ten highly variable regions) were extracted from the cp genomes of 11 species in *Fritillaria* and *Lilium* using *Cardiocrinum giganteum* or *Alstroemeria aurea* as the outgroup for phylogenetic study. Each data set was used to construct phylogenetic trees using the ML, MP and BI analytical methods, and all tree topology structures were nearly identical. Therefore, the phylogenetic studies are presented here using the ML tree with the support values from the MP and BI analyses recorded at the corresponding nodes (Figs 5 & 6).

The phylogenetic tree based on all data sets achieved higher support values. All phylogenetic trees showed that the species of *Fritillaria* clustered into two clades. One monophyletic clade comprised *F. karelinii* (sect. *Rhinopetalum*) and *F. meleagroides* (sect. *Fritillaria*) with strong support ([ML] bootstrap = 100, [MP] bootstrap ≥ 97, [BI] PP ≥ 0.99). The other monophyletic clade was robustly supported ([ML] bootstrap = 100, [MP] bootstrap ≥ 99, [BI] PP = 1) and comprised two subclades: (1) all members of subgenus *Fritillaria* except *F. meleagroides* ([ML] bootstrap = 100, [MP] bootstrap ≥ 99, [BI] PP = 1) and (2) *F. eduardii* (sect. *Petilium*) and *F. persica* (sect. *Theresia*; [ML] bootstrap = 100, [MP] bootstrap ≥ 97, [BI] PP ≥ 0.99). In common with the findings of Day *et al*.^11^, the subgenus *Fritillaria* species were gathered into two large clades, indicating that subgenus *Fritillaria* is a polyphyletic group. Moreover, except that of the IR regions, the topological structures of all data sets were in complete agreement. In the phylogenetic tree based on the IR regions, *F. cirrhosa* and *F. unibracteata* var. *wabuensis* formed a clade with low support ([ML] bootstrap = 41, [MP] bootstrap = 0, [BI] PP = 0.65). However, *F. unibracteata* var. *wabuensis* and *F. taipaiensis* formed a robustly supported clade ([ML] bootstrap ≥ 96, [MP] bootstrap ≥ 86, [BI] PP = 1) in the phylogenetic tree based on other data sets. Because the IR regions were the most conserved, and in other reports, the support values of phylogenetic trees have shown that this region contained insufficient phylogenetic signal^30,48^, the clade *F. cirrhosa* (*F. unibracteata* var. *wabuensis* - *F. taipaiensis*) had a higher reliability. In addition, the phylogenetic tree based on the highly variable regions with higher resolution had the same topological structure as the tree based on the full chloroplast genome sequence, which illustrated that the hot spot regions selected in this study had enormous utility in the study of species identification and phylogeny.

## Conclusions

In this study, the chloroplast genomes of four *Fritillaria* species were reported by de novo sequencing, and comparative genomic analyses with four previously published cp genome sequences of *Fritillaria* were performed. These eight complete cp genomes of *Fritillaria* shared most common genomic features but still provided rich genetic information for the study of *Fritillaria* species in terms of sequence differentiation and structure. SSRs, large repeat sequences and highly variable regions were identified as possible genetic markers. Genetic markers are available for the perfection of plants fingerprints and the identification of similar *Fritillaria* bulbs. Besides, it will be better to realize the genetic relationship of *Fritillaria* species, when the phylogenetic tree is constructed with genetic markers. Not only can phylogenetic tree perfect the classification in *Fritillaria*, but it can also be used to select the parent with nearer genetic relationship and hybridization compatibility in breeding research. This information expands researchers’ horizons regarding the diversity of *Fritillaria* plants and enhances understanding of the phylogenetic relationships among *Fritillaria* species. These data are also valuable for promoting germplasm innovation in *Fritillaria* species, identifying closely related species and cultivars, and protecting useful hereditary phenotypic traits. This study provides a basis for future studies of conservation, breeding, phyletic evolution and population genetics, development of DNA barcodes, and diverse research in *Fritillaria*.

## Materials and Methods

### Sample preparation and sequencing

Fresh leaves of four *Fritillaria* species were sampled (Table S1). Total genomic DNA was isolated from 100 mg of fresh leaf tissue using the DNAsecure Plant Kit (Aidlab, Beijing, China). The quality and concentration of the DNA were measured using agarose gel electrophoresis and a NanoDrop 2000 spectrophotometer (Thermo Fisher Scientific, America).

Illumina paired-end libraries with an average insert size of 500 bp were prepared using the Turbo DNA Library Prep Kit for Illumina (Vazyme) according to the manufacturer’s instructions. Each library was sequenced in a single lane on a HiSeq. 4000 Sequencing System (Illumina, San Diego, California, USA) at Novogene (http://www.novogene.com/index.php), Beijing, China. The raw data were exported for the primary analysis.

### Genome assembly, annotation and analysis

Each of the four *Fritillaria* species was sequenced to produce approximately 1.0–17.0 Gb raw reads, and cp genome reads were extracted by mapping all raw reads to the reference genomes. The high-quality reads were assessed and de novo assembled using SPAdes 3.6.1^49^. After trimming the sequence, all published *Fritillaria* species were used as references to map the contigs with BLAST and thus confirm the plastid genome contigs. The gaps were filled with Sequencher 5.4.6 (http://www.genecodes.com) by PCR amplification and Sanger sequencing.

Gene annotation of the protein-coding genes, transfer RNAs, and ribosomal RNAs was performed with the online program Dual Organellar GenoMe Annotator^50^. From this initial annotation, putative start and stop codons and the exon/intron positions were determined, and then the draft annotation was inspected and corrected manually by comparison with homologous genes in *Fritillaria* and *Lilium* from the NCBI database. Subsequently, the tRNA and rRNA genes were further confirmed by the tRNAscan-SE 1.21^51^ program and BLASTN searches against the same database of plastomes. The schematic diagram of the circular cp genome map was drawn using OGDraw v1.2^52^, with subsequent manual editing.

### Genome comparative analysis

The available complete cp genome sequences of *Fritillaria* including *F. cirrhosa* (KF769143), *F. hupehensis* (KF712486), *F. taipaiensis* (KF769144)^16^ and *F. unibracteata* var. *wabuensis* (KF769142)^53^ were downloaded from GenBank databases (www.ncbi.nlm.nih.gov). These sequences were used for comparison with the complete cp genomes of four *Fritillaria* species acquired in this study. The eight *Fritillaria* cp genomes were divided into four subgenera (Table S2). The multiple sequence alignment of cp genome sequences was performed using MAFFT v7^54^ with the default settings and adjusted manually where necessary within the software BioEdit v7.2.5^55^. The evolutionary divergences of these eight species were evaluated using nucleotide differences and p-distance by MEGA v6 software^56^. DnaSP v5.10^57^ was used to calculate the variable and parsimony-informative sites and nucleotide diversity of four regions (the complete cp genomes, LSC, SSC, and part of the IR regions) of the *Fritillaria* cp genomes. Additionally, the IR expansion/contraction regions were compared among the eight *Fritillaria* species.

### Sequence repeats and SSRs

REPuter^58^, a web-based analysis tool, was used to detect and locate the repeat sequences, including forward (direct), reverse, complement and palindromic (inverted) matching, with a minimal repeat size of 30 bp and more than 90% sequence identity (Hamming distance less than or equal to 3) in the *Fritillaria* species. SSRs were evaluated by the Perl script MISA (http://pgrc.ipk-gatersleben.de/misa/misa.html). The minimum thresholds were set to ten repeat units for mononucleotide SSRs, five repeat units for dinucleotide SSRs, four repeat units for trinucleotide SSRs and three repeat units for tetra-, penta-, and hexanucleotide SSRs. The multiple sequence alignment and visualization of cp genome sequences was performed using MAFFT v7^54^ and Geneious v10.0.6^59^.

### Identification of highly variable regions

Based on the aligned sequence matrix of the cp genomes, the nucleotide variability (Pi) and polymorphic sites (S) were evaluated using a sliding window analysis with a step size of 200 bp and window length of 600 bp in DnaSP v5.10^57^. When the number of polymorphic sites was more than the sum of the average and double the standard deviation, the sequences were extracted as highly variable regions. Then, the locations of these highly variable regions were confirmed using the annotated cp genome. Variable sites, parsimony-informative sites and the nucleotide diversity of the highly variable regions were evaluated by DnaSP v5.10^57^.

### Phylogenetic analyses

Sequences of the eight *Fritillaria* species, three *Lilium* species, *Cardiocrinum giganteum* and *Alstroemeria aurea* were used to perform phylogenetic analysis (Table S2). The analysis was performed based on the following five data sets: (1) the complete chloroplast genome sequence; (2) the LSC region; (3) the SSC region; (4) the IR regions; (5) a concatenation of the sequences of the highly variable regions. The lengths of all alignment matrices of these data sets are shown in Supplementary Table S3. The evolutionary history was deduced using maximum parsimony (MP), maximum likelihood (ML) and Bayesian inference (BI) analysis. For the first four phylogenetic analyses, *Cardiocrinum giganteum* was used as the outgroup. For the phylogenetic trees based on the highly variable regions, *Cardiocrinum giganteum* and *Alstroemeria aurea* were used as the outgroup.

The MP analysis was conducted with PAUP* 4.0b10^60^ using a heuristic search, 1000 random addition sequences, the tree bisection-reconnection (TBR) algorithm and the ‘MulTrees’ option in effect. The MP analysis was performed using the CIPRES Science Gateway v3.3^61^ with RAxML-HPC BlackBox v.8.1.24^62^. Branch support was estimated with 1000 bootstrap replicates in MP and ML analyses. For BI analysis, best substitution models were tested according to the Akaike information criterion (AIC) by MrModeltest2.3^63^ (Table S3), and BI trees were constructed using MrBayes v3.2.2^64^. The Markov Chain Monte Carlo (MCMC) algorithm was calculated for 5,000,000 generations with a sampling of trees every 1000 generations. The first 25% of the generations were discarded as burn-in after checking for stationarity and convergence of the chains, and a consensus tree was constructed using the remaining trees.

## Electronic supplementary material

Supplementary Information
