# Supplementary material for: Chloroplast genomic resources for phylogeny and DNA barcoding: a case study on Fritillaria
Source: Sci Rep. 2018 Jan 19;8:1184. doi: 10.1038/s41598-018-19591-9 (PMC5775360; doi:10.1038/s41598-018-19591-9)
Supplement: Supplementary file 1 — Supplementary Information [file 41598_2018_19591_MOESM1_ESM.pdf]

Chloroplast genomic resources for phylogeny and DNA barcoding: a case study on *Fritillaria*

Yu Bi[1,2,3], Ming-fang Zhang[1,2], Jing Xue[1,2], Ran Dong[3], Yun-peng Du[1,2], Xiu-hai Zhang[1,2]

1 Beijing Agro-Biotechnology Research Center, Beijing Academy of Agriculture and Forestry Sciences, Beijing 100097, P. R. China

2 Beijing Key Laboratory of Agricultural Genetic Resources and Biotechnology, Beijing Engineering Technology Research Center of Functional Floriculture, Beijing 100097, P. R. China

3 Engineering Research Center of Mt. Changbai Ecological Resources Development, Changhcun Sci-Tech University, Changchun 130600, Jilin Province, P. R. China

Yu Bi, Ming-fang Zhang and Jing Xue contributed equally to this work.

*Co-corresponding authors:

Yun-peng Du E-mail: dyp_851212@126.com

Fax: 86-10-51503868

Xiu-hai Zhang, E-mail: zhangxiuhai@baafs.net.cn

Additional Information

Table S1 Sampled species and their voucher specimens used in this study.

Table S2 Accession numbers of chloroplast genome sequences included in phylogenetic analyses.

Table S3 Length of each dataset matrix used for phylogeny construction and the best-fitting models tested by MrModeltest2.3 for Bayesian Inference analyses.

Table S4 List of genes in the chloroplast genome of *Fritillaria*.

Table S5 Distribution of simple sequence repeats (SSRs) loci in the eight *Fritillaria* chloroplast genomes. SSR locus with * in the column of SSR No. indicated that the SSR was same in repeat unit and the number of the repeat unit for the each of e eight *Fritillaria* cp genomes.

Table S6 The number of different repeat units in the eight *Fritillaria* cp genomes.

Table S7 A list of repeated sequences and their locations identified in the eight *Fritillaria* chloroplast genomes.

Table S1. Sampled species and their voucher specimens used in this stu

Species	Subgenus	Voucher	Origin
F. eduardii	*Petilium*	BOP040914	Central Asia
F. karelinii	*Rhinopetalum*	BOP040596	China
F. meleagroides	*Fritinarai*	BOP201959	China
F. persica	*Theresia*	BOP040920	Middle East

Table S2. Accession numbers of chloroplast genome sequences included in phylogenetic analyses.

Species	Subgenus	Genus	Family	Order	Accession No.
F. cirrhosa	*Fritillaria*	*Fritillaria*	Liliaceae	Liliales	KF769143
F. eduardii	*Petilium*	*Fritillaria*	Liliaceae	Liliales	MF947708
F. hupehensis	*Fritillaria*	*Fritillaria*	Liliaceae	Liliales	KF712486
F. karelinii	*Rhinopetalum*	*Fritillaria*	Liliaceae	Liliales	KX354691
F. meleagroides	*Fritillaria*	*Fritillaria*	Liliaceae	Liliales	MF947710
F. persica	*Theresia*	*Fritillaria*	Liliaceae	Liliales	MF947709
F. taipaiensis	*Fritillaria*	*Fritillaria*	Liliaceae	Liliales	KF769144
F. unibracteata var. *wabuensis*	*Fritillaria*	*Fritillaria*	Liliaceae	Liliales	KF769142
Lilium cernuum	*Sinomartagon*	*lilium*	Liliaceae	Liliales	KX354692
Lilium fargesii	*Lophophorum*	*lilium*	Liliaceae	Liliales	KX592156
Lilium taliense	*Sinomartagon*	*lilium*	Liliaceae	Liliales	KY009938
Cardiocrinum giganteum		*Cardiocrinum*	Liliaceae	Liliales	KX528334
Alstroemeria aurea		*Alstroemeria*	Alstroemeriaceae	Liliales	KC968976

Table S3. Length of each dataset matrix used for phylogeny construction and the best-fitting models tested by MrModeltest2.3 for Bayesian Inference analyses.

Dataset	Length (bp)	Selected substitution models
Complete chloroplast genome	156,975	GTR+I+G
LSC	85,542	GTR+I+G
SSC	18,246	GTR+I+G
IR	27,082	GTR+I
hotspot regions sequences (*Cardiocrinum giganteum* as outgroup)	9,220	GTR+I+G
hotspot regions sequences (*Alstroemeria aurea* as outgroup)	9,992	GTR+G

Table S4. List of genes in the chloroplast genome of *Fritillaria*.

Category for genes	Group of gene	Name of gene
Photosynthesis related genes	Photosystem Ⅰ	*psaA, psaB, psaC, psaI, psaJ*
	Photosystem Ⅱ	*psbA, psbB, psbC, psbD, psbE, psbF, psbH, psbI, psbJ, psbK, psbL, psbM, psbN, psbT, psbZ*
	cytochrome b/f compelx	*petA, *petB, *petD, petG, petL, petN*
	ATP synthase	*atpA, atpB, atpE, *atpF, atpH, atpI*
	cytochrome c synthesis	*ccsA*
	Assembly/stability of photosystem Ⅰ	***ycf3,ycf4*
	NADPH dehydrogenase	**ndhA, *ndhB* (x2), *ndhC, ndhD, ndhE, ndhF ,ndhG, ndhH, ndhI, ndhJ, ndhK*
	Rubisco	*rbcL*
Transcription and translation related genes	transcription	*rpoA, rpoB, *rpoC1, rpoC2*
	ribosomal proteins	*rps2, rps3, rps4, rps7* (x2), *rps8, rps11, *rps12* (x2), *rps14,rps15, *rps16, rps18, rps19, *rpl2* (x2), *rpl14, *rpl16, rpl20, rpl22, rpl23* (x2), *rpl32, rpl33,rpl36*
RNA genes	ribosomal RNA	*rrn5* (x2), *rrn4.5* (x2), *rrn16* (x2), *rrn23* (x2)
	transfer RNA	**trnA-UGC* (x2), *trnC-GCA, trnD-GUC, trnE-UUC, trnF-GAA, trnfM-CAU, trnG-GCC, trnG-UCC, trnH-GUG* (x2), *trnI-CAU* (x2), **trnI-GAU* (x2),**trnK-UUU, trnL-CAA* (x2), **trnL-UAA, trnL-UAG, trnM-CAU, trnN-GUU* (x2), *trnP-UGG, trnQ-UUG,trnR-ACG* (x2), *trnR-UCU, trnS-GCU, trnS-GGA, trnS-UGA, trnT-GGU,trnT-UGU, trnV-GAC* (x2), **trnV-UAC, trnW-CCA, trnY-GUA*
Other genes	RNA processing	*matK*
	carbon metabolism	*cemA*
	fatty acid synthesis	*accD*
	proteolysis	***clpP*
Genes of unknown function	conserved reading frames	*ycf1* (x2), *ycf2* (x2)

*: Genes with one intron, **: Gene with two introns, (x2): Gene with duplicated.

Table S5. Distribution of simple sequence repeats (SSRs) loci in the eight *Fritillaria* chloroplast genomes. SSR locus with * in the column of SSR No. indicated that the SSR was same in repeat unit and the number of the repeat unit for the each of e eight *Fritillaria* cp genomes.

Genomes /SSR No.	repeat unit	No. repeat unit	SSR size	Minimum	Maximum	Location	Locus	region
F. cirrhosa								
1	T	13	13	1,678	1,690	*trnK-UUU*	intron	LSC
2	T	11	11	1,948	1,958	*matK*	CDS	LSC
3	A	13	13	4,007	4,019	*trnK-UUU* /*rps16*	IGS	LSC
4	T	17	17	4,188	4,204	*trnK-UUU* /*rps17*	IGS	LSC
5	AT	6	12	5,516	5,527	*rps16*	intron	LSC
6	A	18	18	6,717	6,734	*psbK* /*psbI*	IGS	LSC
7	AT	6	12	7,250	7,261	*trnS-GCU* /*trnG-GCC*	IGS	LSC
8	T	14	14	7,678	7,691	*trnS-GCU* /*trnG-GCC*	IGS	LSC
9	T	15	15	8,049	8,063	*trnG-GCC*	intron	LSC
10	TA	6	12	8,817	8,828	*trnG-GCC* /*atpA*	IGS	LSC
11	T	10	10	8,829	8,838	*trnG-GCC* /*atpA*	IGS	LSC
12	A	14	14	11,654	11,667	*atpF*	intron	LSC
13	T	10	10	12,777	12,786	*atpH* /*atpI*	IGS	LSC
14	A	12	12	13,005	13,016	*atpH* /*atpI*	IGS	LSC
15	ATA	4	12	13,059	13,070	*atpH* /*atpI*	IGS	LSC
16*	AATT	3	12	14,459	14,470	*atpI* /*rps2*	IGS	LSC
17	T	12	12	15,229	15,240	*rps2* /*rpoC2*	IGS	LSC
18*	T	12	12	17,316	17,327	*rpoC2*	CDS	LSC
19	T	11	11	17,425	17,435	*rpoC2*	CDS	LSC
20*	TA	5	10	18,791	18,800	*rpoC2*	CDS	LSC
21	TAAT	3	12	27,854	27,865	*psbM* /*trnD-GUC*	IGS	LSC
22	A	18	18	28,184	28,201	*psbM* /*trnD-GUC*	IGS	LSC
23	T	14	14	28,506	28,519	*trnD-GUC* /*trnY-GUA*	IGS	LSC
24	A	13	13	28,540	28,552	*trnD-GUC* /*trnY-GUA*	IGS	LSC
25	AT	5	10	29,065	29,074	*trnE-UUC* /*trnT-GGU*	IGS	LSC
26	T	17	17	29,348	29,364	*trnE-UUC* /*trnT-GGU*	IGS	LSC
27	A	11	11	29,674	29,684	*trnT-GGU* /*psbD*	IGS	LSC
28	TA	5	10	30,039	30,048	*trnT-GGU* /*psbD*	IGS	LSC
29	AT	5	10	30,208	30,217	*trnT-GGU* /*psbD*	IGS	LSC
30	A	10	10	33,842	33,851	*psbZ* /*trnG-UCC*	IGS	LSC
31	T	11	11	39,525	39,535	*psaA* /*ycf3*	IGS	LSC
32	A	14	14	40,020	40,033	*psaA* /*ycf3*	IGS	LSC
33	A	10	10	42,815	42,824	*trnS-GGA* /*rps4*	IGS	LSC
34	A	12	12	42,860	42,871	*trnS-GGA* /*rps4*	IGS	LSC
35	T	10	10	44,073	44,082	*trnT-UGU* /*trnL-UAA*	IGS	LSC
36	A	19	19	44,420	44,438	*trnT-UGU* /*trnL-UAA*	IGS	LSC
37	A	18	18	44,624	44,641	*trnT-UGU* /*trnL-UAA*	IGS	LSC
38	AT	6	12	45,127	45,138	*trnL-UAA*	intron	LSC
39	T	18	18	45,441	45,458	*trnL-UAA* /*trnF-GAA*	IGS	LSC
40	T	11	11	52,023	52,033	*atpB* /*rbcL*	IGS	LSC
41	A	11	11	56,120	56,130	*accD* /*psaI*	IGS	LSC
42	A	14	14	56,635	56,648	*accD* /*psaI*	IGS	LSC
43*	GAA	4	12	56,670	56,681	*accD* /*psaI*	IGS	LSC

44	A	14	14	58,653	58,666	*cemA*	CDS	LSC
45	T	11	11	62,987	62,997	*psbE* /*petL*	IGS	LSC
46	A	15	15	63,878	63,892	*trnW-CCA* /*trnP-UGG*	IGS	LSC
47	A	11	11	64,581	64,591	*psaJ* /*rpl33*	IGS	LSC
48	A	14	14	64,628	64,641	*psaJ* /*rpl33*	IGS	LSC
49	AT	6	12	64,880	64,891	*psaJ* /*rpl33*	IGS	LSC
50	T	10	10	68,234	68,243	*clpP*	intron	LSC
51	A	11	11	68,621	68,631	*clpP*	intron	LSC
52	T	10	10	68,944	68,953	*clpP*	intron	LSC
53	A	15	15	72,517	72,531	*petB*	intron	LSC
54	AT	7	14	75,415	75,428	*petD* /*rpoA*	IGS	LSC
55*	TTCT	3	12	79,882	79,893	*rpl16*	intron	LSC
56	T	17	17	80,269	80,285	*rpl16* /*rps3*	IGS	LSC
57	A	14	14	81,506	81,519	*rpl22* /*rps19*	intron	LSC
58	T	10	10	82,952	82,961	*rpl2*	intron	IRA
59*	GA	5	10	87,229	87,238	*ycf2*	CDS	IRA
60	T	13	13	111,309	111,321	*rpl32* /*trnL-UAG*	IGS	SSC
61	A	13	13	111,366	111,378	*rpl32* /*trnL-UAG*	IGS	SSC
62*	AATA	3	12	113,147	113,158	*ndhD*	CDS	SSC
63*	A	10	10	115,917	115,926	*ndhG*	CDS	SSC
64	AAAT	4	16	116,262	116,277	*ndhG* /*ndhI*	IGS	SSC
65*	TC	5	10	120,157	120,166	*ndhH*	CDS	SSC
66	T	14	14	121,172	121,185	*rps15* /*ycf1*	IGS	SSC
67	AAAT	3	12	121,226	121,237	*rps15* /*ycf1*	IGS	SSC
68*	T	10	10	121,672	121,681	*ycf1*	CDS	SSC
69	T	15	15	122,287	122,301	*ycf1*	CDS	SSC
70	T	14	14	122,500	122,513	*ycf1*	CDS	SSC
71	T	16	16	122,943	122,958	*ycf1*	CDS	SSC
72*	T	11	11	123,503	123,513	*ycf1*	CDS	SSC
73*	TC	5	10	146,523	146,532	*ycf2*	CDS	IRB
74	A	10	10	150,800	150,809	*rpl2*	intron	IRB
F. eduardii								
1	A	12	12	4,081	4,092	*trnK-UUU* /*rps16*	IGS	LSC
2	T	18	18	4,285	4,302	*trnK-UUU* /*rps17*	IGS	LSC
3	A	20	20	7,162	7,181	*psbK-psbI*	intron	LSC
4	AT	5	10	7,685	7,694	*trnS-GCU* /*trnG-GCC*	IGS	LSC
5	A	18	18	7,951	7,968	*trnS-GCU* /*trnG-GCC*	IGS	LSC
6	T	13	13	8,055	8,067	*trnS-GCU* /*trnG-GCC*	IGS	LSC
7	T	10	10	9,012	9,021	*trnG-GCC* /*trnR-UCU*	IGS	LSC
8	AGAA	3	12	9,069	9,080	*trnG-GCC* /*trnR-UCU*	IGS	LSC
9	TA	6	12	9,188	9,199	*trnG-GCC* /*atpA*	IGS	LSC
10	T	14	14	11,565	11,578	*atpF*	intron	LSC
11	T	15	15	13,132	13,146	*atpH* /*atpI*	IGS	LSC
12	A	10	10	13,364	13,373	*atpH* /*atpI*	IGS	LSC
13*	AATT	3	12	14,810	14,821	*atpI* /*rps2*	IGS	LSC
14*	T	12	12	17,659	17,670	*rpoC2*	CDS	LSC
15	T	11	11	17,768	17,778	*rpoC2*	CDS	LSC
16*	TA	5	10	19,134	19,143	*rpoC2*	CDS	LSC
17	TA	5	10	26,379	26,388	*rpoB* /*trnC-GCA*	IGS	LSC
18	TAAT	3	12	28,153	28,164	*psbM* /*trnD-GUC*	IGS	LSC
19	T	16	16	28,841	28,856	*trnD-GUC* /*trnY-GUA*	IGS	LSC

20	AT	5	10	29,393	29,402	*trnE-UUC* / *trnT-GGU*	IGS	LSC
21	T	13	13	29,618	29,630	*trnE-UUC* / *trnT-GGU*	IGS	LSC
22	A	15	15	29,933	29,947	*trnT-GGU* / *psbD*	IGS	LSC
23	T	15	15	39,761	39,775	*psaA* / *ycf3*	IGS	LSC
24	TTTA	3	12	40,060	40,071	*psaA* / *ycf3*	IGS	LSC
25	TC	5	10	42,057	42,066	*ycf3*	intron	LSC
26	A	11	11	43,087	43,097	*trnS-GGA* / *rps4*	IGS	LSC
27	A	17	17	44,635	44,651	*trnT-UGU* / *trnL-UAA*	IGS	LSC
28	AT	6	12	45,331	45,342	*trnL-UAA*	intron	LSC
29	A	14	14	56,818	56,831	*accD* / *psaI*	IGS	LSC
30*	GAA	4	12	56,853	56,864	*accD* / *psaI*	IGS	LSC
31	A	10	10	58,807	58,816	*cemA*	CDS	LSC
32	T	14	14	63,136	63,149	*psbE* / *petL*	IGS	LSC
33	A	10	10	64,030	64,039	*trnW-CCA* / *trnP-UGG*	IGS	LSC
34	A	11	11	64,738	64,748	*psaJ* / *rpl33*	IGS	LSC
35	T	16	16	68,369	68,384	*clpP*	intron	LSC
36	A	10	10	68,761	68,770	*clpP*	intron	LSC
37	T	10	10	69,083	69,092	*clpP*	intron	LSC
38	A	13	13	72,675	72,687	*petB*	intron	LSC
39	AT	6	12	75,535	75,546	*petD* / *rpoA*	IGS	LSC
40*	TTCT	3	12	80,084	80,095	*rpl16*	intron	LSC
41	T	17	17	80,468	80,484	*rpl16* / *rps3*	IGS	LSC
42	A	15	15	81,717	81,731	*rpl22* / *rps19*	intron	LSC
43*	GA	5	10	87,466	87,475	*ycf2*	CDS	IRA
44	A	16	16	105,968	105,983	*rrn5* / *trnR-ACG*	IGS	IRA
45	A	16	16	110,667	110,682	*ndhF* / *rpl32*	IGS	SSC
46	T	16	16	111,557	111,572	*rpl32* / *trnL-UAG*	IGS	SSC
47*	AATA	3	12	113,394	113,405	*ndhD*	CDS	SSC
48*	A	10	10	116,164	116,173	*ndhG*	CDS	SSC
49	AAAT	4	16	116,509	116,524	*ndhG* / *ndhI*	IGS	SSC
50	AT	6	12	118,406	118,417	*ndhA*	intron	SSC
51*	TC	5	10	120,399	120,408	*ndhH*	CDS	SSC
52	T	20	20	121,413	121,432	*rps15* / *ycf1*	IGS	SSC
53	AAAT	3	12	121,474	121,485	*rps15* / *ycf1*	IGS	SSC
54*	T	10	10	121,920	121,929	*ycf1*	CDS	SSC
55	T	15	15	122,535	122,549	*ycf1*	CDS	SSC
56	T	11	11	122,748	122,758	*ycf1*	CDS	SSC
57*	T	11	11	123,720	123,730	*ycf1*	CDS	SSC
58	A	10	10	124,926	124,935	*ycf1*	CDS	SSC
59	T	16	16	128,233	128,248	*trnR-ACG* / *rrn5*	IGS	IRB
60*	TC	5	10	146,741	146,750	*ycf2*	CDS	IRB
F. hupehensis								
1	A	21	21	4,001	4,021	*trnK-UUU* / *rps16*	IGS	LSC
2	A	10	10	4,038	4,047	*trnK-UUU* / *rps17*	IGS	LSC
3	T	11	11	4,216	4,226	*trnK-UUU* / *rps18*	IGS	LSC
4	A	15	15	6,741	6,755	*psbK-psbI*	intron	LSC
5	AT	6	12	7,271	7,282	*trnS-GCU* / *trnG-GCC*	IGS	LSC
6	T	10	10	7,388	7,397	*trnS-GCU* / *trnG-GCC*	IGS	LSC
7	T	12	12	8,093	8,104	*trnG-GCC*	intron	LSC
8	AGAA	3	12	8,740	8,751	*trnG-GCC*	intron	LSC
9	TA	5	10	8,859	8,868	*trnG-GCC* / *atpA*	IGS	LSC

10	T	10	10	8,869	8,878	*trnG-GCC* / *atpA*	IGS	LSC
11	A	13	13	11,700	11,712	*atpF*	intron	LSC
12	T	10	10	12,823	12,832	*atpH* / *atpI*	IGS	LSC
13	A	14	14	13,051	13,064	*atpH* / *atpI*	IGS	LSC
14	ATA	4	12	13,107	13,118	*atpH* / *atpI*	IGS	LSC
15*	AATT	3	12	14,515	14,526	*atpI* / *rps2*	IGS	LSC
16	T	12	12	15,285	15,296	*rps2* / *rpoC2*	IGS	LSC
17*	T	12	12	17,371	17,382	*rpoC2*	CDS	LSC
18	T	11	11	17,480	17,490	*rpoC2*	CDS	LSC
19*	TA	5	10	18,846	18,855	*rpoC2*	CDS	LSC
20	TAAT	3	12	27,901	27,912	*psbM* / *trnD-GUC*	IGS	LSC
21	A	12	12	28,274	28,285	*psbM* / *trnD-GUC*	IGS	LSC
22	T	12	12	28,596	28,607	*trnD-GUC* / *trnY-GUA*	IGS	LSC
23	AT	5	10	29,151	29,160	*trnE-UUC* / *trnT-GGU*	IGS	LSC
24	T	10	10	29,329	29,338	*trnE-UUC* / *trnT-GGU*	IGS	LSC
25	T	14	14	29,440	29,453	*trnE-UUC* / *trnT-GGU*	IGS	LSC
26	A	15	15	29,760	29,774	*trnT-GGU* / *psbD*	IGS	LSC
27	TA	5	10	30,130	30,139	*trnT-GGU* / *psbD*	IGS	LSC
28	AT	5	10	30,299	30,308	*trnT-GGU* / *psbD*	IGS	LSC
29	A	10	10	33,941	33,950	*psbZ* / *trnG-UCC*	IGS	LSC
30	T	10	10	39,625	39,634	*psaA* / *ycf3*	IGS	LSC
31	T	10	10	39,811	39,820	*psaA* / *ycf3*	IGS	LSC
32	TTTA	3	12	39,921	39,932	*psaA* / *ycf3*	IGS	LSC
33	A	10	10	40,128	40,137	*psaA* / *ycf3*	IGS	LSC
34	A	10	10	41,887	41,896	*ycf3*	intron	LSC
35	TC	5	10	41,927	41,936	*ycf3*	intron	LSC
36	A	10	10	41,960	41,969	*ycf3*	intron	LSC
37	A	12	12	42,930	42,941	*trnS-GGA* / *rps4*	IGS	LSC
38	T	10	10	44,181	44,190	*trnT-UGU* / *trnL-UAA*	IGS	LSC
39	ATTAT	3	15	44,394	44,408	*trnT-UGU* / *trnL-UAA*	IGS	LSC
40	A	11	11	44,530	44,540	*trnT-UGU* / *trnL-UAA*	IGS	LSC
41	A	13	13	44,725	44,737	*trnT-UGU* / *trnL-UAA*	IGS	LSC
42	AT	6	12	45,223	45,234	*trnL-UAA*	intron	LSC
43	T	11	11	45,537	45,547	*trnL-UAA* / *trnF-GAA*	IGS	LSC
44	A	11	11	56,230	56,240	*accD* / *psaI*	IGS	LSC
45*	GAA	4	12	56,773	56,784	*accD* / *psaI*	IGS	LSC
46	A	17	17	58,721	58,737	*cemA*	CDS	LSC
47	AT	5	10	61,225	61,234	*petA* / *psbJ*	IGS	LSC
48	A	16	16	62,945	62,960	*psbE* / *petL*	IGS	LSC
49	T	10	10	63,067	63,076	*psbE* / *petL*	IGS	LSC
50	T	13	13	63,091	63,103	*psbE* / *petL*	IGS	LSC
51	A	13	13	63,984	63,996	*trnW-CCA* / *trnP-UGG*	IGS	LSC
52	A	18	18	64,730	64,747	*psaJ* / *rpl33*	IGS	LSC
53	TA	5	10	64,938	64,947	*psaJ* / *rpl33*	IGS	LSC
54	AT	5	10	64,990	64,999	*psaJ* / *rpl33*	IGS	LSC
55	T	14	14	68,344	68,357	*clpP*	intron	LSC
56	A	11	11	68,735	68,745	*clpP*	intron	LSC
57	T	14	14	69,057	69,070	*clpP*	intron	LSC
58	A	15	15	72,633	72,647	*petB*	intron	LSC
59	T	12	12	73,076	73,087	*petB*	intron	LSC
60	AT	6	12	75,527	75,538	*petD* / *rpoA*	IGS	LSC

61*	TTCT	3	12	80,011	80,022	*rpl16*	intron	LSC
62	T	10	10	80,394	80,403	*rpl16* /*rps3*	IGS	LSC
63	A	15	15	81,629	81,643	*rpl22* /*rps19*	intron	LSC
64	T	11	11	83,077	83,087	*rpl2*	intron	IRA
65*	GA	5	10	87,355	87,364	*ycf2*	CDS	IRA
66	A	14	14	105,855	105,868	*rrn5* /*trnR-ACG*	IGS	IRA
67	T	14	14	111,457	111,470	*rpl32* /*trnL-UAG*	IGS	SSC
68*	AATA	3	12	113,292	113,303	*ndhD*	CDS	SSC
69	ATACA	3	15	115,172	115,186	*psaC* /*ndhE*	IGS	SSC
70*	A	10	10	116,067	116,076	*ndhG*	CDS	SSC
71	AAAT	4	16	116,412	116,427	*ndhG* /*ndhI*	IGS	SSC
72	A	12	12	116,725	116,736	*ndhG* /*ndhI*	IGS	SSC
73	A	10	10	118,167	118,176	*ndhA*	intron	SSC
74*	TC	5	10	120,308	120,317	*ndhH*	CDS	SSC
75	A	10	10	120,790	120,799	*ndhH* /*rps15*	IGS	SSC
76	T	11	11	121,324	121,334	*rps15* /*ycf1*	IGS	SSC
77	AAAT	3	12	121,375	121,386	*rps15* /*ycf1*	IGS	SSC
78*	T	10	10	121,821	121,830	*ycf1*	CDS	SSC
79	T	15	15	122,436	122,450	*ycf1*	CDS	SSC
80	T	14	14	122,649	122,662	*ycf1*	CDS	SSC
81	T	16	16	123,092	123,107	*ycf1*	CDS	SSC
82*	T	11	11	123,652	123,662	*ycf1*	CDS	SSC
83	T	14	14	128,172	128,185	*trnR-ACG* /*rrn5*	IGS	IRB
84*	TC	5	10	146,676	146,685	*ycf2*	CDS	IRB
85	A	11	11	150,953	150,963	*rpl2*	intron	IRB
F. karelinii								
1	T	12	12	1,688	1,699	*trnK-UUU*	intron	LSC
2	A	10	10	4,076	4,085	*trnK-UUU* /*rps16*	IGS	LSC
3	T	23	23	4,283	4,305	*trnK-UUU* /*rps17*	IGS	LSC
4	A	14	14	4,689	4,702	*trnK-UUU* /*rps18*	IGS	LSC
5	A	16	16	7,180	7,195	*psbK-psbI*	intron	LSC
6	CA	5	10	7,196	7,205	*psbK-psbI*	intron	LSC
7	AT	6	12	7,701	7,712	*trnS-GCU* /*trnG-GCC*	IGS	LSC
8	A	13	13	8,144	8,156	*trnS-GCU* /*trnG-GCC*	IGS	LSC
9	AGAA	3	12	9,155	9,166	*trnS-GCU* /*trnG-GCC*	IGS	LSC
10	TA	5	10	9,274	9,283	*trnG-GCC* /*atpA*	IGS	LSC
11	T	10	10	9,284	9,293	*trnG-GCC* /*atpA*	IGS	LSC
12	A	13	13	12,098	12,110	*atpF*	intron	LSC
13	T	12	12	13,219	13,230	*atpH* /*atpI*	IGS	LSC
14	A	14	14	13,443	13,456	*atpH* /*atpI*	IGS	LSC
15*	AATT	3	12	14,930	14,941	*atpI* /*rps2*	IGS	LSC
16*	T	12	12	17,792	17,803	*rpoC2*	CDS	LSC
17	T	11	11	17,901	17,911	*rpoC2*	CDS	LSC
18*	TA	5	10	19,267	19,276	*rpoC2*	CDS	LSC
19	TTTA	3	12	21,923	21,934	*rpoC1*	intron	LSC
20	TA	5	10	26,506	26,515	*rpoB* /*trnC-GCA*	IGS	LSC
21	T	10	10	27,747	27,756	*psbM* /*trnD-GUC*	IGS	LSC
22	T	16	16	28,970	28,985	*trnD-GUC* /*trnY-GUA*	IGS	LSC
23	A	13	13	30,053	30,065	*trnT-GGU* /*psbD*	IGS	LSC
24	CAAA	3	12	33,588	33,599	*psbC* /*trnS-UGA*	IGS	LSC
25	A	12	12	42,135	42,146	*ycf3*	intron	LSC

26	TC	5	10	42,174	42,183	*ycf3*	intron	LSC
27	A	12	12	43,176	43,187	*trnS-GGA* /*rps4*	IGS	LSC
28	A	12	12	43,223	43,234	*trnS-GGA* /*rps4*	IGS	LSC
29	T	18	18	43,944	43,961	*rps4* /*trnT-UGU*	IGS	LSC
30	A	23	23	44,736	44,758	*trnT-UGU* /*trnL-UAA*	IGS	LSC
31	AT	5	10	45,447	45,456	*trnT-UGU* /*trnL-UAA*	intron	LSC
32	T	15	15	45,756	45,770	*trnL-UAA* /*trnF-GAA*	IGS	LSC
33*	GAA	4	12	56,973	56,984	*accD* /*psaI*	IGS	LSC
34	A	11	11	58,927	58,937	*cemA*	CDS	LSC
35	TAAA	3	12	63,164	63,175	*psbE* /*petL*	IGS	LSC
36	T	10	10	64,581	64,590	*trnP-UGG* /*psaJ*	IGS	LSC
37	T	13	13	68,221	68,233	*clpP*	intron	LSC
38	A	11	11	68,615	68,625	*clpP*	intron	LSC
39	T	10	10	68,939	68,948	*clpP*	intron	LSC
40	A	11	11	72,524	72,534	*petB*	intron	LSC
41	ATTA	3	12	78,140	78,151	*rps8* /*rpl14*	IGS	LSC
42*	TTCT	3	12	79,956	79,967	*rpl16*	intron	LSC
43	T	12	12	80,356	80,367	*rpl16* /*rps3*	IGS	LSC
44	A	21	21	81,605	81,625	*rpl22* /*rps19*	intron	LSC
45*	GA	5	10	87,368	87,377	*ycf2*	CDS	IRA
46	A	16	16	105,861	105,876	*rrn5* /*trnR-ACG*	IGS	IRA
47	A	16	16	110,586	110,601	*ndhF* /*rpl32*	IGS	SSC
48	TA	5	10	110,625	110,634	*ndhF* /*rpl32*	IGS	SSC
49	T	11	11	111,495	111,505	*rpl32* /*trnL-UAG*	IGS	SSC
50	A	11	11	111,568	111,578	*rpl32* /*trnL-UAG*	IGS	SSC
51*	AATA	3	12	113,346	113,357	*ndhD*	CDS	SSC
52*	A	10	10	116,115	116,124	*ndhG*	CDS	SSC
53	AAAT	3	12	116,460	116,471	*ndhG* /*ndhI*	IGS	SSC
54	A	11	11	116,759	116,769	*ndhG* /*ndhI*	IGS	SSC
55*	TC	5	10	120,331	120,340	*ndhH*	CDS	SSC
56*	T	10	10	121,776	121,785	*ycf1*	CDS	SSC
57	T	16	16	122,397	122,412	*ycf1*	CDS	SSC
58*	T	11	11	123,584	123,594	*ycf1*	CDS	SSC
59	A	10	10	124,784	124,793	*ycf1*	CDS	SSC
60	T	16	16	128,118	128,133	*trnR-ACG* /*rrn5*	IGS	IRB
61*	TC	5	10	146,617	146,626	*ycf2*	CDS	IRB
F. meleagroides								
1	A	21	21	3,980	4,000	*trnK-UUU* /*rps16*	IGS	LSC
2	T	10	10	4,181	4,190	*trnK-UUU* /*rps16*	IGS	LSC
3	T	10	10	6,032	6,041	*rps16* /*trnQ-UUG*	IGS	LSC
4	A	19	19	7,049	7,067	*psbK-psbI*	intron	LSC
5	AT	6	12	7,572	7,583	*trnS-GCU* /*trnG-GCC*	IGS	LSC
6	A	20	20	7,840	7,859	*trnS-GCU* /*trnG-GCC*	IGS	LSC
7	AGAA	3	12	9,006	9,017	*trnS-GCU* /*trnG-GCC*	IGS	LSC
8	TA	7	14	9,125	9,138	*trnG-GCC* /*atpA*	IGS	LSC
9	T	14	14	11,525	11,538	*atpF*	intron	LSC
10	A	14	14	11,976	11,989	*atpF*	intron	LSC
11	A	10	10	12,953	12,962	*atpH* /*atpI*	IGS	LSC
12	T	16	16	13,096	13,111	*atpH* /*atpI*	IGS	LSC
13	A	16	16	13,328	13,343	*atpH* /*atpI*	IGS	LSC
14*	AATT	3	12	14,816	14,827	*atpI* /*rps2*	IGS	LSC

15	T	16	16	15,586	15,601	*rps2* / *rpoC2*	IGS	LSC
16*	T	12	12	17,692	17,703	*rpoC2*	CDS	LSC
17	T	15	15	17,801	17,815	*rpoC2*	CDS	LSC
18*	TA	5	10	19,167	19,176	*rpoC2*	CDS	LSC
19	T	11	11	20,200	20,210	*rpoC1*	CDS	LSC
20	TTTA	3	12	21,823	21,834	*rpoC1*	intron	LSC
21	TTTAC	3	15	21,962	21,976	*rpoC1*	intron	LSC
22	T	10	10	27,654	27,663	*psbM* / *trnD-GUC*	IGS	LSC
23	TAAT	3	12	28,216	28,227	*psbM* / *trnD-GUC*	IGS	LSC
24	T	15	15	28,895	28,909	*trnD-GUC* / *trnY-GUA*	IGS	LSC
25	AT	5	10	29,452	29,461	*trnE-UUC* / *trnT-GGU*	IGS	LSC
26	T	17	17	29,691	29,707	*trnE-UUC* / *trnT-GGU*	IGS	LSC
27	AT	5	10	29,804	29,813	*trnE-UUC* / *trnT-GGU*	IGS	LSC
28	A	15	15	30,013	30,027	*trnT-GGU* / *psbD*	IGS	LSC
29	TA	5	10	30,379	30,388	*trnT-GGU* / *psbD*	IGS	LSC
30	AT	5	10	30,529	30,538	*trnT-GGU* / *psbD*	IGS	LSC
31	A	12	12	34,157	34,168	*psbZ* / *trnG-UCC*	IGS	LSC
32	T	21	21	39,844	39,864	*psaA* / *ycf3*	IGS	LSC
33	T	14	14	40,044	40,057	*psaA* / *ycf3*	IGS	LSC
34	TTTA	3	12	40,158	40,169	*psaA* / *ycf3*	IGS	LSC
35	A	15	15	42,106	42,120	*ycf3*	intron	LSC
36	TC	5	10	42,148	42,157	*ycf3*	intron	LSC
37	A	10	10	43,138	43,147	*trnS-GGA* / *rps4*	IGS	LSC
38	A	12	12	43,183	43,194	*trnS-GGA* / *rps4*	IGS	LSC
39	TAT	4	12	44,663	44,674	*trnT-UGU* / *trnL-UAA*	IGS	LSC
40	A	17	17	44,738	44,754	*trnT-UGU* / *trnL-UAA*	IGS	LSC
41	A	16	16	44,936	44,951	*trnT-UGU* / *trnL-UAA*	IGS	LSC
42	T	24	24	45,756	45,779	*trnL-UAA* / *trnF-GAA*	IGS	LSC
43	T	10	10	46,422	46,431	*trnF-GAA* / *ndhJ*	IGS	LSC
44	A	19	19	56,631	56,649	*accD* / *psaI*	IGS	LSC
45	A	23	23	56,915	56,937	*accD* / *psaI*	IGS	LSC
46*	GAA	4	12	56,960	56,971	*accD* / *psaI*	IGS	LSC
47	A	19	19	58,910	58,928	*cemA*	CDS	LSC
48	T	12	12	63,239	63,250	*psbE* / *petL*	IGS	LSC
49	A	16	16	64,161	64,176	*trnW-CCA* / *trnP-UGG*	IGS	LSC
50	T	11	11	64,557	64,567	*trnP-UGG* / *psaJ*	IGS	LSC
51	AT	5	10	65,160	65,169	*psaJ* / *rpl33*	IGS	LSC
52	T	16	16	68,514	68,529	*clpP*	intron	LSC
53	A	20	20	68,912	68,931	*clpP*	intron	LSC
54	T	10	10	69,244	69,253	*clpP*	intron	LSC
55	A	12	12	69,922	69,933	*clpP* / *psbB*	IGS	LSC
56	A	11	11	72,543	72,553	*psbH* / *petB*	IGS	LSC
57	A	23	23	72,834	72,856	*petB*	intron	LSC
58	AT	5	10	75,731	75,740	*petD* / *rpoA*	IGS	LSC
59	AT	5	10	75,749	75,758	*petD* / *rpoA*	IGS	LSC
60	AT	5	10	75,764	75,773	*petD* / *rpoA*	IGS	LSC
61*	TTCT	3	12	80,282	80,293	*rpl16*	intron	LSC
62	T	12	12	80,357	80,368	*rpl16*	intron	LSC
63	A	18	18	81,900	81,917	*rpl22* / *rps19*	intron	LSC
64	T	10	10	83,351	83,360	*rpl2*	intron	IRA
65*	GA	5	10	87,628	87,637	*ycf2*	CDS	IRA

66	A	16	16	106,113	106,128	*rrn5 /trnR-ACG*	IGS	IRA
67	AT	5	10	110,846	110,855	*ndhF /rpl32*	IGS	SSC
68	T	16	16	111,687	111,702	*rpl32 /trnL-UAG*	IGS	SSC
69	A	18	18	111,742	111,759	*rpl32 /trnL-UAG*	IGS	SSC
70*	AATA	3	12	113,538	113,549	*ndhD*	CDS	SSC
71*	A	10	10	116,307	116,316	*ndhG*	CDS	SSC
72	AAAT	4	16	116,652	116,667	*ndhG /ndhI*	IGS	SSC
73	A	11	11	118,139	118,149	*ndhA*	intron	SSC
74*	TC	5	10	120,286	120,295	*ndhH*	CDS	SSC
75*	T	10	10	121,734	121,743	*ycf1*	CDS	SSC
76	T	12	12	122,355	122,366	*ycf1*	CDS	SSC
77	T	14	14	122,513	122,526	*ycf1*	CDS	SSC
78	T	10	10	122,956	122,965	*ycf1*	CDS	SSC
79*	T	11	11	123,510	123,520	*ycf1*	CDS	SSC
80	T	16	16	127,980	127,995	*trnR-ACG /rrn5*	IGS	IRB
81*	TC	5	10	146,471	146,480	*ycf2*	CDS	IRB
82	A	10	10	150,748	150,757	*rpl2*	intron	IRB
F. persica								
1	T	15	15	1,678	1,692	*trnK-UUU*	intron	LSC
2	A	11	11	4,006	4,016	*trnK-UUU /rps16*	IGS	LSC
3	T	23	23	4,216	4,238	*trnK-UUU /rps16*	IGS	LSC
4	A	14	14	6,753	6,766	*psbK-psbI*	intron	LSC
5	AT	5	10	7,270	7,279	*trnS-GCU /trnG-GCC*	IGS	LSC
6	T	15	15	8,592	8,606	*trnG-GCC /trnR-UCU*	IGS	LSC
7	TA	5	10	8,769	8,778	*trnG-GCC /atpA*	IGS	LSC
8	T	10	10	8,779	8,788	*trnG-GCC /atpA*	IGS	LSC
9	A	11	11	11,610	11,620	*atpF*	intron	LSC
10	T	13	13	12,730	12,742	*atpH /atpI*	IGS	LSC
11	A	12	12	12,961	12,972	*atpH /atpI*	IGS	LSC
12*	AATT	3	12	14,427	14,438	*atpI /rps2*	IGS	LSC
13	T	18	18	15,197	15,214	*rps2 /rpoC2*	IGS	LSC
14*	T	12	12	17,290	17,301	*rpoC2*	CDS	LSC
15	T	11	11	17,399	17,409	*rpoC2*	CDS	LSC
16*	TA	5	10	18,771	18,780	*rpoC2*	CDS	LSC
17	TAAT	3	12	27,809	27,820	*psbM /trnD-GUC*	IGS	LSC
18	T	14	14	28,503	28,516	*trnD-GUC /trnY-GUA*	IGS	LSC
19	A	13	13	29,576	29,588	*trnT-GGU /psbD*	IGS	LSC
20	A	10	10	30,458	30,467	*trnT-GGU /psbD*	IGS	LSC
21	T	17	17	39,431	39,447	*psaA /ycf3*	IGS	LSC
22	TTTA	4	16	39,732	39,747	*psaA /ycf3*	IGS	LSC
23	A	13	13	41,688	41,700	*ycf3*	intron	LSC
24	TC	5	10	41,728	41,737	*ycf3*	intron	LSC
25	A	15	15	42,759	42,773	*trnS-GGA /rps4*	IGS	LSC
26	A	23	23	44,336	44,358	*trnT-UGU /trnL-UAA*	IGS	LSC
27	AT	5	10	45,038	45,047	*trnL-UAA*	intron	LSC
28*	GAA	4	12	56,591	56,602	*accD /psaI*	IGS	LSC
29	A	12	12	58,545	58,556	*cemA*	CDS	LSC
30	T	14	14	62,879	62,892	*psbE /petL*	IGS	LSC
31	A	14	14	63,773	63,786	*trnW-CCA /trnP-UGG*	IGS	LSC
32	A	15	15	64,495	64,509	*psaJ /rpl33*	IGS	LSC
33	A	11	11	64,546	64,556	*psaJ /rpl33*	IGS	LSC

34	AT	5	10	64,789	64,798	*psaJ* /*rpl33*	IGS	LSC
35	T	11	11	68,145	68,155	*clpP*	intron	LSC
36	T	15	15	68,853	68,867	*clpP*	intron	LSC
37	A	13	13	72,432	72,444	*petB*	intron	LSC
38	T	13	13	72,873	72,885	*petB*	intron	LSC
39	TAT	4	12	75,339	75,350	*petD* /*rpoA*	IGS	LSC
40	ATTA	3	12	78,055	78,066	*rps8* /*rpl14*	IGS	LSC
41*	TTCT	3	12	79,735	79,746	*rpl16*	intron	LSC
42	T	13	13	80,123	80,135	*rpl16* /*rps3*	IGS	LSC
43*	GA	5	10	87,088	87,097	*ycf2*	CDS	IRA
44	A	13	13	105,562	105,574	*rrn5* /*trnR-ACG*	IGS	IRA
45	A	13	13	110,277	110,289	*ndhF* /*rpl32*	IGS	SSC
46	A	11	11	111,216	111,226	*rpl32* /*trnL-UAG*	IGS	SSC
47*	AATA	3	12	112,996	113,007	*ndhD*	CDS	SSC
48*	A	10	10	115,766	115,775	*ndhG*	CDS	SSC
49	AAAT	4	16	116,111	116,126	*ndhG* /*ndhI*	IGS	SSC
50	TAAA	3	12	116,432	116,443	*ndhG* /*ndhI*	IGS	SSC
51	AT	6	12	117,999	118,010	*ndhA*	intron	SSC
52*	TC	5	10	119,993	120,002	*ndhH*	CDS	SSC
53	AAAT	3	12	121,057	121,068	*rps15* /*ycf1*	IGS	SSC
54*	T	10	10	121,503	121,512	*ycf1*	CDS	SSC
55	T	15	15	122,118	122,132	*ycf1*	CDS	SSC
56	T	11	11	122,331	122,341	*ycf1*	CDS	SSC
57	T	10	10	122,774	122,783	*ycf1*	CDS	SSC
58*	T	11	11	123,328	123,338	*ycf1*	CDS	SSC
59	T	12	12	124,011	124,022	*ycf1*	CDS	SSC
60	ATT	4	12	124,700	124,711	*ycf1*	CDS	SSC
61	T	13	13	127,864	127,876	*trnR-ACG* /*rrn5*	IGS	IRB
62*	TC	5	10	146,341	146,350	*ycf2*	CDS	IRB
F. taipaiensis								
1	T	12	12	1,678	1,689	*trnK-UUU*	intron	LSC
2	A	16	16	4,005	4,020	*trnK-UUU* /*rps16*	IGS	LSC
3	T	14	14	4,214	4,227	*trnK-UUU* /*rps16*	IGS	LSC
4	A	19	19	6,726	6,744	*psbK-psbI*	intron	LSC
5	AT	7	14	7,260	7,273	*trnS-GCU* /*trnG-GCC*	IGS	LSC
6	AT	6	12	7,277	7,288	*trnS-GCU* /*trnG-GCC*	IGS	LSC
7	T	12	12	7,705	7,716	*trnS-GCU* /*trnG-GCC*	IGS	LSC
8	T	13	13	8,074	8,086	*trnG-GCC*	intron	LSC
9	AGAA	3	12	8,721	8,732	*trnG-GCC*	intron	LSC
10	TA	6	12	8,840	8,851	*trnG-GCC* /*atpA*	IGS	LSC
11	A	13	13	11,669	11,681	*atpF*	intron	LSC
12	T	10	10	12,786	12,795	*atpH* /*atpI*	IGS	LSC
13	A	15	15	13,014	13,028	*atpH* /*atpI*	IGS	LSC
14*	AATT	3	12	14,460	14,471	*atpI* /*rps2*	IGS	LSC
15	T	17	17	15,230	15,246	*rps2* /*rpoC2*	IGS	LSC
16*	T	12	12	17,323	17,334	*rpoC2*	CDS	LSC
17	T	11	11	17,432	17,442	*rpoC2*	CDS	LSC
18*	TA	5	10	18,798	18,807	*rpoC2*	CDS	LSC
19	TAAT	3	12	27,824	27,835	*psbM* /*trnD-GUC*	IGS	LSC
20	A	20	20	28,158	28,177	*psbM* /*trnD-GUC*	IGS	LSC
21	T	12	12	28,482	28,493	*trnD-GUC* /*trnY-GUA*	IGS	LSC

22	A	11	11	28,517	28,527	*trnD-GUC* /*trnY-GUA*	IGS	LSC
23	AT	5	10	29,039	29,048	*trnE-UUC* /*trnT-GGU*	IGS	LSC
24	T	17	17	29,317	29,333	*trnE-UUC* /*trnT-GGU*	IGS	LSC
25	TATTA	3	15	29,442	29,456	*trnE-UUC* /*trnT-GGU*	IGS	LSC
26	A	11	11	29,648	29,658	*trnT-GGU* /*psbD*	IGS	LSC
27	TA	5	10	29,851	29,860	*trnT-GGU* /*psbD*	IGS	LSC
28	AT	5	10	30,020	30,029	*trnT-GGU* /*psbD*	IGS	LSC
29	A	12	12	33,654	33,665	*psbZ* /*trnG-UCC*	IGS	LSC
30	T	10	10	39,340	39,349	*psaA* /*ycf3*	IGS	LSC
31	A	16	16	39,834	39,849	*psaA* /*ycf3*	IGS	LSC
32	A	11	11	41,593	41,603	*ycf3*	intron	LSC
33	TC	5	10	41,639	41,648	*ycf3*	intron	LSC
34	A	11	11	42,630	42,640	*trnS-GGA* /*rps4*	IGS	LSC
35	A	11	11	42,676	42,686	*trnS-GGA* /*rps4*	IGS	LSC
36	A	13	13	44,254	44,266	*trnT-UGU* /*trnL-UAA*	IGS	LSC
37	A	15	15	44,454	44,468	*trnT-UGU* /*trnL-UAA*	IGS	LSC
38	AT	6	12	44,954	44,965	*trnL-UAA*	intron	LSC
39	T	12	12	45,268	45,279	*trnL-UAA* /*trnF-GAA*	IGS	LSC
40	A	10	10	55,935	55,944	*accD* /*psaI*	IGS	LSC
41	A	16	16	56,312	56,327	*accD* /*psaI*	IGS	LSC
42*	GAA	4	12	56,349	56,360	*accD* /*psaI*	IGS	LSC
43	A	15	15	58,297	58,311	*cemA*	CDS	LSC
44	AT	5	10	60,799	60,808	*petA* /*psbJ*	IGS	LSC
45	A	16	16	62,514	62,529	*psbE* /*petL*	IGS	LSC
46	T	12	12	62,639	62,650	*psbE* /*petL*	IGS	LSC
47	A	15	15	63,531	63,545	*trnW-CCA* /*trnP-UGG*	IGS	LSC
48	A	10	10	64,234	64,243	*psaJ* /*rpl33*	IGS	LSC
49	A	14	14	64,280	64,293	*psaJ* /*rpl33*	IGS	LSC
50	T	15	15	67,891	67,905	*clpP*	intron	LSC
51	A	11	11	68,283	68,293	*clpP*	intron	LSC
52	T	15	15	68,606	68,620	*clpP*	intron	LSC
53	A	14	14	72,183	72,196	*petB*	intron	LSC
54	AT	8	16	75,075	75,090	*petD* /*rpoA*	IGS	LSC
55*	TTCT	3	12	79,545	79,556	*rpl16*	intron	LSC
56	T	10	10	79,618	79,627	*rpl16*	intron	LSC
57	T	12	12	79,930	79,941	*rpl16* /*rps3*	IGS	LSC
58	A	16	16	81,171	81,186	*rpl22* /*rps19*	intron	LSC
59	T	11	11	82,620	82,630	*rpl2*	intron	IRA
60*	GA	5	10	86,898	86,907	*ycf2*	CDS	IRA
61	A	10	10	110,102	110,111	*ndhF* /*rpl32*	IGS	SSC
62	AAT	5	15	110,731	110,745	*rpl32* /*trnL-UAG*	IGS	SSC
63	T	13	13	110,992	111,004	*rpl32* /*trnL-UAG*	IGS	SSC
64	A	16	16	111,049	111,064	*rpl32* /*trnL-UAG*	IGS	SSC
65*	AATA	3	12	112,831	112,842	*ndhD*	CDS	SSC
66*	A	10	10	115,601	115,610	*ndhG*	CDS	SSC
67	AAAT	4	16	115,946	115,961	*ndhG* /*ndhI*	IGS	SSC
68	A	12	12	116,259	116,270	*ndhG* /*ndhI*	IGS	SSC
69*	TC	5	10	119,848	119,857	*ndhH*	CDS	SSC
70	T	14	14	120,863	120,876	*rps15* /*ycf1*	IGS	SSC
71*	AAAT	3	12	120,918	120,929	*rps15* /*ycf1*	IGS	SSC
72	T	10	10	121,364	121,373	*ycf1*	CDS	SSC

73	T	15	15	121,979	121,993	*ycf1*	CDS	SSC
74	T	14	14	122,192	122,205	*ycf1*	CDS	SSC
75	T	16	16	122,635	122,650	*ycf1*	CDS	SSC
76*	T	11	11	123,195	123,205	*ycf1*	CDS	SSC
77*	TC	5	10	146,222	146,231	*ycf2*	CDS	IRB
78	A	11	11	150,499	150,509	*rpl2*	intron	IRB
F. unibracteata var. *wabuensis*								
1	T	15	15	1,679	1,693	*trnK-UUU*	intron	LSC
2	T	10	10	3,596	3,605	*trnK-UUU*	intron	LSC
3	A	16	16	4,011	4,026	*trnK-UUU/rps16*	IGS	LSC
4	A	10	10	4,043	4,052	*trnK-UUU/rps16*	IGS	LSC
5	T	16	16	4,221	4,236	*trnK-UUU/rps16*	IGS	LSC
6	T	15	15	6,082	6,096	*rps16/trnQ-UUG*	IGS	LSC
7	A	18	18	6,759	6,776	*psbK-psbI*	intron	LSC
8	AT	6	12	7,225	7,236	*trnS-GCU/trnG-GCC*	IGS	LSC
9	T	15	15	7,668	7,682	*trnS-GCU/trnG-GCC*	IGS	LSC
10	AGAA	3	12	8,686	8,697	*trnS-GCU/trnG-GCC*	IGS	LSC
11	T	11	11	8,813	8,823	*trnG-GCC/atpA*	IGS	LSC
12	A	16	16	12,979	12,994	*atpH/atpI*	IGS	LSC
13*	AATT	3	12	14,435	14,446	*atpI/rps2*	IGS	LSC
14	T	15	15	15,205	15,219	*rps2/rpoC2*	IGS	LSC
15*	T	12	12	17,295	17,306	*rpoC2*	CDS	LSC
16	T	11	11	17,404	17,414	*rpoC2*	CDS	LSC
17*	TA	5	10	18,770	18,779	*rpoC2*	CDS	LSC
18	TAAT	3	12	27,807	27,818	*psbM/trnD-GUC*	IGS	LSC
19	A	19	19	28,141	28,159	*psbM/trnD-GUC*	IGS	LSC
20	T	16	16	28,464	28,479	*trnD-GUC/trnY-GUA*	IGS	LSC
21	A	10	10	28,500	28,509	*trnD-GUC/trnY-GUA*	IGS	LSC
22	AT	5	10	29,024	29,033	*trnE-UUC/trnT-GGU*	IGS	LSC
23	T	16	16	29,301	29,316	*trnE-UUC/trnT-GGU*	IGS	LSC
24	A	13	13	29,626	29,638	*trnT-GGU/psbD*	IGS	LSC
25	TA	5	10	29,995	30,004	*trnT-GGU/psbD*	IGS	LSC
26	AT	5	10	30,173	30,182	*trnT-GGU/psbD*	IGS	LSC
27	A	11	11	33,807	33,817	*psbZ/trnG-UCC*	IGS	LSC
28	T	12	12	39,493	39,504	*psaA/ycf3*	IGS	LSC
29	A	15	15	39,989	40,003	*psaA/ycf3*	IGS	LSC
30	A	14	14	41,752	41,765	*ycf3*	intron	LSC
31	TC	5	10	41,800	41,809	*ycf3*	intron	LSC
32	A	13	13	42,791	42,803	*trnS-GGA/rps4*	IGS	LSC
33	T	15	15	44,006	44,020	*trnT-UGU/trnL-UAA*	IGS	LSC
34	A	18	18	44,393	44,410	*trnT-UGU/trnL-UAA*	IGS	LSC
35	A	17	17	44,594	44,610	*trnT-UGU/trnL-UAA*	IGS	LSC
36	AT	6	12	45,096	45,107	*trnL-UAA*	intron	LSC
37	T	15	15	45,410	45,424	*trnL-UAA/trnF-GAA*	IGS	LSC
38	A	16	16	56,089	56,104	*accD/psaI*	IGS	LSC
39	A	11	11	56,617	56,627	*accD/psaI*	IGS	LSC
40*	GAA	4	12	56,649	56,660	*accD/psaI*	IGS	LSC
41	A	14	14	58,161	58,174	*cemA*	CDS	LSC
42	AT	5	10	60,680	60,689	*petA/psbJ*	IGS	LSC
43	T	12	12	62,503	62,514	*psbE/petL*	IGS	LSC
44	A	19	19	63,396	63,414	*trnW-CCA/trnP-UGG*	IGS	LSC

45	A	11	11	64,108	64,118	*psaJ* /*rpl33*	IGS	LSC
46	A	12	12	64,155	64,166	*psaJ* /*rpl33*	IGS	LSC
47	T	13	13	67,759	67,771	*clpP*	intron	LSC
48	A	11	11	68,150	68,160	*clpP*	intron	LSC
49	T	10	10	68,474	68,483	*clpP*	intron	LSC
50	A	10	10	72,047	72,056	*petB*	intron	LSC
51	AT	7	14	74,929	74,942	*petD* /*rpoA*	IGS	LSC
52*	TTCT	3	12	79,398	79,409	*rpl16*	intron	LSC
53	T	10	10	79,471	79,480	*rpl16*	intron	LSC
54	T	14	14	79,775	79,788	*rpl16* /*rps3*	IGS	LSC
55	A	20	20	81,016	81,035	*rpl22* /*rps19*	intron	LSC
56*	GA	5	10	86,746	86,755	*ycf2*	CDS	IRA
57	A	11	11	107,172	107,182	*ycf1*	intron	IRA/SSC
58	A	11	11	109,690	109,700	*ndhF* /*rpl32*	IGS	SSC
59	T	15	15	110,575	110,589	*rpl32* /*trnL-UAG*	IGS	SSC
60	A	17	17	110,631	110,647	*rpl32* /*trnL-UAG*	IGS	SSC
61*	AATA	3	12	112,413	112,424	*ndhD*	CDS	SSC
62*	A	10	10	115,183	115,192	*ndhG*	CDS	SSC
63	AAAT	4	16	115,528	115,543	*ndhG* /*ndhI*	IGS	SSC
64	A	13	13	115,841	115,853	*ndhG* /*ndhI*	IGS	SSC
65*	TC	5	10	119,424	119,433	*ndhH*	CDS	SSC
66	T	16	16	120,439	120,454	*rps15* /*ycf1*	IGS	SSC
67	AAAT	3	12	120,496	120,507	*rps15* /*ycf1*	IGS	SSC
68*	T	10	10	120,942	120,951	*ycf1*	CDS	SSC
69	T	15	15	121,557	121,571	*ycf1*	CDS	SSC
70	T	14	14	121,770	121,783	*ycf1*	CDS	SSC
71	T	16	16	122,213	122,228	*ycf1*	CDS	SSC
72*	T	11	11	122,773	122,783	*ycf1*	CDS	SSC
73*	TC	5	10	145,542	145,551	*ycf2*	CDS	IRB
74	A	11	11	149,817	149,827	*rpl2*	intron	IRB

Table S6. The number of different repeat units in the eight *Fritillaria* cp genomes.

Species	mono-	di-			tri-		tetra-				penta-			Total
	A/T	AC/ GT	AG/ CT	AT/ AT	AAG/ CTT	AAT/ ATT	AAAC/ GTTT	AAAG/ CTTT	AAAT/ ATTT	AATT/ AATT	AAAGT/ ACTTT	AATAC/ ATTGT	AATAT/ ATATT	
F. karelinii	40	1	4	6	1	0	1	2	4	2	0	0	0	61
F. eduardii	39	0	4	8	1	0	0	2	4	2	0	0	0	60
F. persica	40	0	4	6	1	2	0	1	5	3	0	0	0	62
F. meleagroides	55	0	4	12	1	1	0	2	4	2	1	0	0	82
F. hupehensis	58	0	4	11	1	1	0	2	4	2	0	1	1	85
F. unibracteata var. *wabuensis*	54	0	4	8	1	0	0	2	3	2	0	0	0	74
F. cirrhosa	53	0	3	10	1	1	0	1	3	2	0	0	0	74
F. taipaiensis	54	0	4	10	1	1	0	2	3	2	0	0	1	78
Total	393	1	31	71	8	6	1	14	30	17	1	1	2	576
	393 (68.23%)	103 (17.88%)			14 (2.43%)		62 (10.76%)				4 (0.69%)			

Table S7. A list of repeated sequences and their locations identified in the eight *Fritillaria* chloroplast genomes. CIR, EDU, HUP, KAR, MEL, PER, TAI, UNI represented *F. cirrhosa* , *F. eduaidii* , *F. hupehensi* , *F. karelinii* , *F. meleagroides* , *F. persica* , *F. taipaiensis* and *F. unibracteata* var. *wabuensis* , respectively.

Genomes /repeats No.	Repeat Type	Length (bp)	Position A	Locus	Region	Position B	Locus	Region	Shared genomes
F. cirrhosa									
1	P	65	27058	*petN-psbM*	LSC	27058	*petN-psbM*	LSC	ALL
2	F	57	88946	*ycf2*	IRA	88970	*ycf2*	IRA	CIR, HUP, MEL, TAI, UNI
3	P	57	88946	*ycf2*	IRA	144733	*ycf2*	IRB	CIR, HUP, MEL, TAI, UNI
4	P	57	88970	*ycf2*	IRA	144757	*ycf2*	IRB	CIR, HUP, MEL, TAI, UNI
5	F	57	144733	*ycf2*	IRB	144757	*ycf2*	IRB	CIR, HUP, MEL, TAI, UNI
6	F	50	36289	*psaB*	LSC	38513	*psaA*	LSC	ALL
7	P	38	26834	*trnC-GCA-petN*	LSC	26834	*trnC-GCA-petN*	LSC	ALL
8	P	34	112386	*ccsA*	SSC	112386	*ccsA*	SSC	ALL
9	F	37	144753	*ycf2*	IRB	144777	*ycf2*	IRB	CIR, HUP, TAI, UNI
10	F	39	41176	*ycf3*	LSC	96349	*rps12-trnV-GAC*	IRA	CIR, EDU, HUP, KAR, PER, TAI, UNI
11	P	39	41176	*ycf3*	LSC	137373	*trnV-GAC-rps12*	IRB	CIR, EDU, HUP, KAR, PER, TAI, UNI
12	P	35	91828	*ycf2-trnL-CAA*	IRA	91828	*ycf2-trnL-CAA*	IRA	ALL
13	F	35	91828	*ycf2-trnL-CAA*	IRA	141897	*trnL-CAA-ycf2*	IRB	ALL
14	P	35	141897	*trnL-CAA-ycf2*	IRB	141897	*trnL-CAA-ycf2*	IRB	ALL
15	F	30	58453	*ycf4-cemA*	LSC	58482	*ycf4-cemA*	LSC	CIR
16	P	33	44158	*trnT-UGU-trnL-UAA*	LSC	44158	*trnT-UGU-trnL-UAA*	LSC	CIR, EDU, HUP, PER, TAI, UNI
17	F	38	30182	*trnT-GGU-psbD*	LSC	30195	*trnT-GGU-psbD*	LSC	CIR, TAI
18	F	37	88946	*ycf2*	IRA	88994	*ycf2*	IRA	CIR, EDU, HUP, TAI, UNI
19	P	37	88946	*ycf2*	IRA	144729	*ycf2*	IRB	CIR, EDU, HUP, TAI, UNI
20	P	37	88994	*ycf2*	IRA	144777	*ycf2*	IRB	CIR, EDU, HUP, TAI, UNI
21	F	37	144729	*ycf2*	IRB	144777	*ycf2*	IRB	CIR, EDU, HUP, TAI, UNI
22	P	34	5345	*rbcL*	LSC	5345	*rbcL*	LSC	CIR, EDU, TAI, UNI
23	F	31	144762	*ycf2*	IRB	144786	*ycf2*	IRB	CIR, HUP, TAI, UNI
24	P	30	112807	*ccsA-ndhD*	SSC	112850	*ccsA-ndhD*	SSC	CIR, EDU, HUP, KAR, PER, TAI, UNI
25	F	32	36307	*psaB*	LSC	38531	*psaA*	LSC	CIR
26	P	31	7018	*psbI-trnS-GCU, trnS-GCU*	LSC	42618	*trnS-GGA*	LSC	ALL
27	R	31	78124	*rps8-rpl14*	LSC	78124	*rps8-rpl14*	LSC	CIR, TAI, UNI

28	R	31	112932	*ccsA-ndhD, ndhD*	SSC	112932	*ccsA-ndhD, ndhD*	SSC	CIR, EDU, HUP, KAR, MEL, TAI, UNI
29	R	30	45434	*trnL-UAA-trnF-GAA*	LSC	45434	*trnL-UAA-trnF-GAA*	LSC	CIR, MEL
30	P	32	33269	*psbC-trnS-UGA, trnS-UGA*	LSC	42620	*trnS-GGA, trnS-GGA-rps4*	LSC	ALL
31	F	32	88975	*ycf2*	IRA	88999	*ycf2*	IRA	CIR, HUP, TAI, UNI
32	P	32	88975	*ycf2*	IRA	144729	*ycf2*	IRB	CIR, HUP, TAI, UNI
33	P	32	88999	*ycf2*	IRA	144753	*ycf2*	IRB	CIR, HUP, TAI, UNI
34	F	31	7016	*psbI-trnS-GCU, trnS-GCU*	LSC	33270	*psbC-trnS-UGA, trnS-UGA*	LSC	ALL
35	C	31	44036	*trnT-UGU-trnL-UAA*	LSC	64843	*psaJ-rpl33*	LSC	CIR, EDU, HUP, MEL, PER, TAI, UNI
36	F	30	8557	*trnG-GCC, trnG-GCC-trnR-UCU*	LSC	34070	*trnG-UCC, trnG-UCC-trnfM-CAU*	LSC	ALL
37	F	30	86606	*ycf2*	IRA	86627	*ycf2*	IRA	ALL
38	P	30	86606	*ycf2*	IRA	147103	*ycf2*	IRB	ALL
39	P	30	86627	*ycf2*	IRA	147124	*ycf2*	IRB	ALL
40	F	30	147106	*ycf2*	IRB	147127	*ycf2*	IRB	ALL
F. eduardii									
1	F	75	79409	*rpl16*	LSC	79478	*rpl16*	LSC	EDU
2	P	65	27391	*petN-psbM*	LSC	27391	*petN-psbM*	LSC	ALL
3	F	59	3807	*matK-trnK-UUU*	LSC	3866	*matK-trnK-UUU*	LSC	EDU
4	P	53	27809	*psbM-trnD-GUC*	LSC	28038	*psbM-trnD-GUC*	LSC	EDU
5	F	48	86822	*ycf2*	IRA	86843	*ycf2*	IRA	EDU
6	P	48	86822	*ycf2*	IRA	147324	*ycf2*	IRB	EDU
7	P	48	86843	*ycf2*	IRA	147345	*ycf2*	IRB	EDU
8	F	48	147324	*ycf2*	IRB	147345	*ycf2*	IRB	EDU
9	F	50	36525	*psaB*	LSC	38749	*psaA*	LSC	ALL
10	P	38	27167	*trnC-GCA-petN*	LSC	27167	*trnC-GCA-petN*	LSC	ALL
11	F	34	1630	*trnK-UUU-matK*	LSC	1658	*trnK-UUU-matK*	LSC	EDU
12	P	34	112633	*ccsA*	SSC	112633	*ccsA*	SSC	ALL
13	F	39	41419	*ycf3*	LSC	96577	*rps12-trnV-GAC*	IRA	CIR, EDU, HUP, KAR, PER, TAI, UNI
14	P	39	41419	*ycf3*	LSC	137599	*trnV-GAC-rps12*	IRB	CIR, EDU, HUP, KAR, PER, TAI, UNI
15	P	35	92036	*ycf2-trnL-CAA*	IRA	92036	*ycf2-trnL-CAA*	IRA	ALL
16	F	35	92036	*ycf2-trnL-CAA*	IRA	142144	*trnL-CAA-ycf2*	IRB	ALL
17	P	35	142144	*trnL-CAA-ycf2*	IRB	142144	*trnL-CAA-ycf2*	IRB	ALL
18	F	37	89183	*ycf2*	IRA	89207	*ycf2*	IRA	CIR, EDU, HUP, TAI, UNI
19	P	37	89183	*ycf2*	IRA	144971	*ycf2*	IRB	CIR, EDU, HUP, TAI, UNI
20	P	37	89207	*ycf2*	IRA	144995	*ycf2*	IRB	CIR, EDU, HUP, TAI, UNI
21	F	37	144971	*ycf2*	IRB	144995	*ycf2*	IRB	CIR, EDU, HUP, TAI, UNI
22	P	33	44390	*trnT-UGU-trnL-UAA*	LSC	44390	*trnT-UGU-trnL-UAA*	LSC	CIR, EDU, HUP, PER, TAI, UNI
23	P	34	5448	*rps16*	LSC	5448	*rps16*	LSC	CIR, EDU, TAI, UNI
24	P	31	7462	*psbI-trnS-GCU, trnS-GCU*	LSC	42850	*trnS-GGA*	LSC	ALL
25	R	31	113179	*ccsA-ndhD, ndhD*	SSC	113179	*ccsA-ndhD, ndhD*	SSC	CIR, EDU, HUP, KAR, MEL, TAI, UNI

26	F	30	36548	*psaB*	LSC	38772	*psaA*	LSC	EDU, HUP, KAR, MEL, PER, TAI, UNI
27	P	30	113056	*ccsA-ndhD*	SSC	113095	*ccsA-ndhD*	SSC	CIR, EDU, HUP, KAR, PER, TAI, UNI
28	P	32	33508	*psbC-trnS-UGA, trnS-UGA*	LSC	42852	*trnS-GGA, trnS-GGA-rps4*	LSC	ALL
29	F	31	7460	*psbI-trnS-GCU*	LSC	33509	*psbC-trnS-UGA, trnS-UGA*	LSC	ALL
30	C	31	44261	*trnT-UGU-trnL-UAA*	LSC	64980	*psaJ-rpl33*	LSC	CIR, EDU, HUP, MEL, PER, TAI, UNI
31	F	30	8926	*trnG-GCC, trnG-GCC-trnR-UCU*	LSC	34308	*trnG-UCC, trnG-UCC-trnfM-CAU*	LSC	ALL
32	R	30	81710	*rpl22-rps19*	LSC	110657	*ndhF-rpl32*	SSC	EDU
33	F	30	86822	*ycf2*	IRA	86864	*ycf2*	IRA	ALL
34	P	30	86822	*ycf2*	IRA	147321	*ycf2*	IRB	ALL
35	P	30	86864	*ycf2*	IRA	147363	*ycf2*	IRB	ALL
36	F	30	147324	*ycf2*	IRB	147366	*ycf2*	IRB	ALL
F. hupehensis									
1	P	65	27106	*petN-psbM*	LSC	27106	*petN-psbM*	LSC	ALL
2	F	57	89072	*ycf2*	IRA	89096	*ycf2*	IRA	CIR, HUP, MEL, TAI, UNI
3	P	57	89072	*ycf2*	IRA	144886	*ycf2*	IRB	CIR, HUP, MEL, TAI, UNI
4	P	57	89096	*ycf2*	IRA	144910	*ycf2*	IRB	CIR, HUP, MEL, TAI, UNI
5	F	57	144886	*ycf2*	IRB	144910	*ycf2*	IRB	CIR, HUP, MEL, TAI, UNI
6	F	50	36389	*psaB*	LSC	38613	*psaA*	LSC	ALL
7	P	38	26882	*trnC-GCA-petN*	LSC	26882	*trnC-GCA-petN*	LSC	ALL
8	P	34	112531	*ccsA*	SSC	112531	*ccsA*	SSC	ALL
9	F	37	144906	*ycf2*	IRB	144930	*ycf2*	IRB	CIR, HUP, TAI, UNI
10	F	39	41281	*ycf3*	LSC	96468	*rps12-trnV-GAC*	IRA	CIR, EDU, HUP, KAR, PER, TAI, UNI
11	P	39	41281	*ycf3*	LSC	137532	*trnV-GAC-rps12*	IRB	CIR, EDU, HUP, KAR, PER, TAI, UNI
12	P	35	91948	*ycf2-trnL-CAA*	IRA	91948	*ycf2-trnL-CAA*	IRA	ALL
13	F	35	91948	*ycf2-trnL-CAA*	IRA	142056	*trnL-CAA-ycf2*	IRB	ALL
14	P	35	142056	*trnL-CAA-ycf2*	IRB	142056	*trnL-CAA-ycf2*	IRB	ALL
15	P	30	112954	*ccsA-ndhD*	SSC	112993	*ccsA-ndhD*	SSC	CIR, EDU, HUP, KAR, PER, TAI, UNI
16	P	33	44266	*trnT-UGU-trnL-UAA*	LSC	44266	*trnT-UGU-trnL-UAA*	LSC	CIR, EDU, HUP, PER, TAI, UNI
17	F	33	78237	*rps8-rpl14*	LSC	78256	*rps8-rpl14*	LSC	HUP
18	F	37	89072	*ycf2*	IRA	89120	*ycf2*	IRA	CIR, EDU, HUP, TAI, UNI
19	P	37	89072	*ycf2*	IRA	144882	*ycf2*	IRB	CIR, EDU, HUP, TAI, UNI
20	P	37	89120	*ycf2*	IRA	144930	*ycf2*	IRB	CIR, EDU, HUP, TAI, UNI
21	F	37	144882	*ycf2*	IRB	144930	*ycf2*	IRB	CIR, EDU, HUP, TAI, UNI
22	F	31	110606	*rpl32-trnL-UAG*	SSC	110624	*rpl32-trnL-UAG*	SSC	HUP
23	F	31	144915	*ycf2*	IRB	144939	*ycf2*	IRB	CIR, HUP, TAI, UNI
24	P	31	7039	*psbI-trnS-GCU, trnS-GCU*	LSC	42732	*trnS-GGA*	LSC	ALL
25	R	31	113077	*ccsA-ndhD, ndhD*	SSC	113077	*ccsA-ndhD, ndhD*	SSC	CIR, EDU, HUP, KAR, MEL, TAI, UNI
26	F	30	30273	*trnT-GGU-psbD*	LSC	30286	*trnT-GGU-psbD*	LSC	HUP
27	F	30	36412	*psaB*	LSC	38636	*psaA*	LSC	EDU, HUP, KAR, MEL, PER, TAI, UNI
28	P	32	33368	*psbC-trnS-UGA, trnS-UGA*	LSC	42734	*trnS-GGA, trnS-GGA-rps4*	LSC	ALL
29	F	32	89101	*ycf2*	IRA	89125	*ycf2*	IRA	CIR, HUP, TAI, UNI
30	P	32	89101	*ycf2*	IRA	144882	*ycf2*	IRB	CIR, HUP, TAI, UNI

31	P	32	89125	*ycf2*	IRA	144906	*ycf2*	IRB	CIR, HUP, TAI, UNI
32	F	31	7037	*psbI-trnS-GCU, trnS-GCU*	LSC	33369	*psbC-trnS-UGA, trnS-UGA*	LSC	ALL
33	C	31	44148	*trnT-UGU-trnL-UAA*	LSC	64953	*psaJ-rpl33*	LSC	CIR, EDU, HUP, MEL, PER, TAI, UNI
34	F	31	65027	*psaJ-rpl33*	LSC	78250	*rps8-rpl14*	LSC	HUP
35	F	30	8599	*trnG-GCC, trnG-GCC-trnR-UCU*	LSC	34169	*trnG-UCC, trnG-UCC-trnfM-CAU*	LSC	ALL
36	F	30	86732	*ycf2*	IRA	86753	*ycf2*	IRA	ALL
37	P	30	86732	*ycf2*	IRA	147256	*ycf2*	IRB	ALL
38	P	30	86753	*ycf2*	IRA	147277	*ycf2*	IRB	ALL
39	F	30	147259	*ycf2*	IRB	147280	*ycf2*	IRB	ALL
F. karelinii									
1	P	65	27521	*petN-psbM*	LSC	27521	*petN-psbM*	LSC	ALL
2	F	56	79256	*rpl16*	LSC	79303	*rpl16*	LSC	KAR
3	F	50	36644	*psaB*	LSC	38868	*psaA*	LSC	ALL
4	P	38	27297	*trnC-GCA-petN*	LSC	27297	*trnC-GCA-petN*	LSC	ALL
5	F	34	86707	*ycf2*	IRA	86731	*ycf2*	IRA	KAR
6	P	34	86707	*ycf2*	IRA	147228	*ycf2*	IRB	KAR
7	P	34	86731	*ycf2*	IRA	147252	*ycf2*	IRB	KAR
8	P	34	112585	*ccsA*	SSC	112585	*ccsA*	SSC	ALL
9	F	34	147228	*ycf2*	IRB	147252	*ycf2*	IRB	KAR
10	F	39	41526	*ycf3*	LSC	96476	*rps12-trnV-GAC*	IRA	CIR, EDU, HUP, KAR, PER, TAI, UNI
11	P	39	41526	*ycf3*	LSC	137478	*trnV-GAC-rps12*	IRB	CIR, EDU, HUP, KAR, PER, TAI, UNI
12	P	35	91935	*ycf2-trnL-CAA*	IRA	91935	*ycf2-trnL-CAA*	IRA	ALL
13	F	35	91935	*ycf2-trnL-CAA*	IRA	142023	*trnL-CAA-ycf2*	IRB	ALL
14	P	35	142023	*trnL-CAA-ycf2*	IRB	142023	*trnL-CAA-ycf2*	IRB	ALL
15	P	30	113008	*ccsA-ndhD*	SSC	113047	*ccsA-ndhD*	SSC	CIR, EDU, HUP, KAR, PER, TAI, UNI
16	R	32	81597	*rpl22-rps19, rps19*	LSC	81600	*rpl22-rps19, rps19*	LSC	KAR
17	P	36	75374	*petD-rpoA*	LSC	75402	*petD-rpoA*	LSC	KAR
18	F	30	1626	*trnK-UUU-matK*	LSC	1654	*trnK-UUU-matK*	LSC	KAR
19	F	32	62627	*petL*	LSC	62652	*petL*	LSC	KAR
20	R	31	4278	*trnK-UUU-rps16*	LSC	4278	*trnK-UUU-rps16*	LSC	KAR, PER
21	P	31	7479	*psbI-trnS-GCU, trnS-GCU*	LSC	42978	*trnS-GGA*	LSC	ALL
22	R	31	113131	*ccsA-ndhD, ndhD*	SSC	113131	*ccsA-ndhD, ndhD*	SSC	CIR, EDU, HUP, KAR, MEL, TAI, UNI
23	P	33	75383	*petD-rpoA*	LSC	75383	*petD-rpoA*	LSC	KAR
24	F	33	89085	*ycf2*	IRA	89109	*ycf2*	IRA	KAR, MEL, PER
25	P	33	89085	*ycf2*	IRA	144851	*ycf2*	IRB	KAR, MEL, PER
26	P	33	89109	*ycf2*	IRA	144875	*ycf2*	IRB	KAR, MEL, PER
27	F	33	144851	*ycf2*	IRB	144875	*ycf2*	IRB	KAR, MEL, PER
28	P	30	7901	*trnS-GCU-trnG-GCC*	LSC	7901	*trnS-GCU-trnG-GCC*	LSC	KAR
29	F	30	36667	*psaB*	LSC	38891	*psaA*	LSC	EDU, HUP, KAR, MEL, PER, TAI, UNI
30	P	32	33625	*psbC-trnS-UGA, trnS-UGA*	LSC	42980	*trnS-GGA, trnS-GGA-rps4*	LSC	ALL
31	F	31	7477	*psbI-trnS-GCU, trnS-GCU*	LSC	33626	*psbC-trnS-UGA, trnS-UGA*	LSC	ALL
32	F	30	9020	*trnG-GCC, trnG-GCC-trnR-UCU*	LSC	34424	*trnG-UCC, trnG-UCC-trnfM-CAU*	LSC	ALL

33	R	30	44735	*trnT-UGU-trnL-UAA*	LSC	110568	*ndhF-rpl32*	SSC	KAR
34	F	30	86745	*ycf2*	IRA	86766	*ycf2*	IRA	ALL
35	P	30	86745	*ycf2*	IRA	147197	*ycf2*	IRB	ALL
36	P	30	86766	*ycf2*	IRA	147218	*ycf2*	IRB	ALL
37	F	30	147200	*ycf2*	IRB	147221	*ycf2*	IRB	ALL
F. meleagroides									
1	P	65	27428	*petN-psbM*	LSC	27428	*petN-psbM*	LSC	ALL
2	P	69	113200	*ccsA-ndhD*	SSC	113200	*ccsA-ndhD*	SSC	MEL
3	F	57	89345	*ycf2*	IRA	89369	*ycf2*	IRA	CIR, HUP, MEL, TAI, UNI
4	P	57	89345	*ycf2*	IRA	144681	*ycf2*	IRB	CIR, HUP, MEL, TAI, UNI
5	P	57	89369	*ycf2*	IRA	144705	*ycf2*	IRB	CIR, HUP, MEL, TAI, UNI
6	F	57	144681	*ycf2*	IRB	144705	*ycf2*	IRB	CIR, HUP, MEL, TAI, UNI
7	F	46	58142	*ycf4, ycf4-cemA*	LSC	58513	*ycf4-cemA*	LSC	MEL
8	F	50	36608	*psaB*	LSC	38832	*psaA*	LSC	ALL
9	P	38	27204	*trnC-GCA-petN*	LSC	27204	*trnC-GCA-petN*	LSC	ALL
10	F	42	79080	*rpl14-rpl16*	LSC	79116	*rpl14-rpl16, rpl16*	LSC	MEL
11	F	41	41507	*ycf3*	LSC	96727	*rps7-trnV-GAC*	IRA	MEL
12	P	41	41507	*ycf3*	LSC	137339	*trnV-GAC-rps12*	IRB	MEL
13	P	34	112777	*ccsA*	SSC	112777	*ccsA*	SSC	ALL
14	F	33	89345	*ycf2*	IRA	89393	*ycf2*	IRA	KAR, MEL, PER
15	P	33	89345	*ycf2*	IRA	144681	*ycf2*	IRB	KAR, MEL, PER
16	P	33	89393	*ycf2*	IRA	144729	*ycf2*	IRB	KAR, MEL, PER
17	F	33	144681	*ycf2*	IRB	144729	*ycf2*	IRB	KAR, MEL, PER
18	P	35	92206	*ycf2-trnL-CAA*	IRA	92206	*ycf2-trnL-CAA*	IRA	ALL
19	F	35	92206	*ycf2-trnL-CAA*	IRA	141866	*trnL-CAA-ycf2*	IRB	ALL
20	P	35	141866	*trnL-CAA-ycf2*	IRB	141866	*trnL-CAA-ycf2*	IRB	ALL
21	F	36	115507	*psaC-ndhE*	SSC	115554	*psaC-ndhE*	SSC	MEL
22	C	30	45752	*trnL-UAA-trnF-GAA*	LSC	56910	*accD-psaI*	LSC	MEL
23	R	32	45752	*trnL-UAA-trnF-GAA*	LSC	45752	*trnL-UAA-trnF-GAA*	LSC	MEL
24	F	34	7033	*psbK-psbI*	LSC	81883	*rpl22-rps19*	LSC	MEL
25	P	31	7350	*psbI-trnS-GCU, trnS-GCU*	LSC	42940	*trnS-GGA*	LSC	ALL
26	R	31	113323	*ccsA-ndhD, ndhD*	SSC	113323	*ccsA-ndhD, ndhD*	SSC	CIR, EDU, HUP, KAR, MEL, TAI, UNI
27	R	30	3970	*trnK-UUU-rps16*	LSC	56916	*accD-psaI*	LSC	MEL
28	F	30	36631	*psaB*	LSC	38855	*psaA*	LSC	EDU, HUP, KAR, MEL, PER, TAI, UNI
29	C	30	39840	*psaA-ycf3*	LSC	56913	*accD-psaI*	LSC	MEL
30	R	30	45751	*trnL-UAA-trnF-GAA*	LSC	45751	*trnL-UAA-trnF-GAA*	LSC	CIR, MEL
31	P	32	33584	*psbC-trnS-UGA, trnS-UGA*	LSC	42942	*trnS-GGA, trnS-GGA-rps4*	LSC	ALL
32	P	32	45754	*trnL-UAA-trnF-GAA*	LSC	56908	*accD-psaI*	LSC	MEL
33	F	32	80348	*rps3-rpl22*	LSC	123501	*ycf1*	SSC	MEL
34	R	31	3979	*trnK-UUU-rps16*	LSC	56904	*accD-psaI*	LSC	MEL
35	F	31	7348	*psbI-trnS-GCU, trnS-GCU*	LSC	33585	*psbC-trnS-UGA, trnS-UGA*	LSC	ALL
36	C	31	44352	*trnT-UGU-trnL-UAA*	LSC	65122	*psaJ-rpl33*	LSC	CIR, EDU, HUP, MEL, PER, TAI, UNI

37	F	31	45745	*trnL-UAA-trnF-GAA*	LSC	45750	*trnL-UAA-trnF-GAA*	LSC	MEL
38	R	31	56920	*accD-psaI*	LSC	58897	*ycf4-cemA, cemA*	LSC	MEL
39	F	31	115522	*psaC-ndhE*	SSC	115569	*psaC-ndhE*	SSC	MEL
40	C	30	3977	*trnK-UUU-rps16*	LSC	45756	*trnL-UAA-trnF-GAA*	LSC	MEL
41	R	30	3979	*trnK-UUU-rps16*	LSC	56906	*accD-psaI*	LSC	MEL
42	F	30	7044	*psbK-psbI*	LSC	7836	*trnS-GCU-trnG-GCC*	LSC	MEL
43	F	30	7839	*trnS-GCU-trnG-GCC*	LSC	7841	*trnS-GCU-trnG-GCC*	LSC	MEL, UNI
44	F	30	8863	*trnG-GCC, trnG-GCC-trnR-UCU*	LSC	34388	*trnG-UCC, trnG-UCC-trnfM-CAU*	LSC	ALL
45	F	30	39840	*psaA-ycf3*	LSC	45752	*trnL-UAA-trnF-GAA*	LSC	MEL
46	P	30	45750	*trnL-UAA-trnF-GAA*	LSC	56913	*accD-psaI*	LSC	MEL
47	P	30	45753	*trnL-UAA-trnF-GAA*	LSC	58897	*ycf4-cemA, cemA*	LSC	MEL
48	R	30	56627	*accD-psaI*	LSC	81891	*rpl22-rps19, rps19*	LSC	MEL
49	R	30	56918	*accD-psaI*	LSC	111729	*rpl32-trnL-UAG*	SSC	MEL, TAI
50	F	30	87005	*ycf2*	IRA	87026	*ycf2*	IRA	ALL
51	P	30	87005	*d*	IRA	147051	*ycf2*	IRB	ALL
52	P	30	87026	*ycf2*	IRA	147072	*ycf2*	IRB	ALL
53	F	30	147054	*ycf2*	IRB	147075	*ycf2*	IRB	ALL
F. persica									
1	P	65	27022	*petN-psbM*	LSC	27022	*petN-psbM*	LSC	ALL
2	F	50	36195	*psaB*	LSC	38419	*psaA*	LSC	ALL
3	P	38	26798	*trnC-GCA-petN*	LSC	26798	*trnC-GCA-petN*	LSC	ALL
4	P	34	112234	*ccsA*	SSC	112234	*ccsA*	SSC	ALL
5	F	39	41093	*ycf3*	LSC	96172	*rps12-trnV-GAC*	IRA	CIR, EDU, HUP, KAR, PER, TAI, UNI
6	P	39	41093	*ycf3*	LSC	137226	*trnV-GAC-rps12*	IRB	CIR, EDU, HUP, KAR, PER, TAI, UNI
7	P	35	91631	*ycf2-trnL-CAA*	IRA	91631	*ycf2-trnL-CAA*	IRA	ALL
8	F	35	91631	*ycf2-trnL-CAA*	IRA	141771	*trnL-CAA-ycf2*	IRB	ALL
9	P	35	141771	*trnL-CAA-ycf2*	IRB	141771	*trnL-CAA-ycf2*	IRB	ALL
10	F	30	12990	*atpH-atpI*	LSC	13014	*atpH-atpI*	LSC	PER
11	P	30	112657	*ccsA-ndhD*	SSC	112696	*ccsA-ndhD*	SSC	CIR, EDU, HUP, KAR, PER, TAI, UNI
12	P	33	44069	*trnT-UGU-trnL-UAA*	LSC	44069	*trnT-UGU-trnL-UAA*	LSC	CIR, EDU, HUP, PER, TAI, UNI
13	R	31	4211	*trnK-UUU-rps16*	LSC	4211	*trnK-UUU-rps16*	LSC	KAR, PER
14	P	31	7047	*psbI-trnS-GCU, trnS-GCU*	LSC	42522	*trnS-GGA*	LSC	ALL
15	F	33	88805	*ycf2*	IRA	88829	*ycf2*	IRA	KAR, MEL, PER
16	P	33	88805	*ycf2*	IRA	144575	*ycf2*	IRB	KAR, MEL, PER
17	P	33	88829	*ycf2*	IRA	144599	*ycf2*	IRB	KAR, MEL, PER
18	F	33	144575	*ycf2*	IRB	144599	*ycf2*	IRB	KAR, MEL, PER
19	F	30	36218	*psaB*	LSC	38442	*psaA*	LSC	EDU, HUP, KAR, MEL, PER, TAI, UNI
20	P	30	120911	*rps15-ycf1*	SSC	120944	*rps15-ycf1*	SSC	PER
21	P	32	33170	*psbC-trnS-UGA, trnS-UGA*	LSC	42524	*trnS-GGA, trnS-GGA-rps4*	LSC	ALL
22	F	31	7045	*psbI-trnS-GCU, trnS-GCU*	LSC	33171	*psbC-trnS-UGA, trnS-UGA*	LSC	ALL
23	C	31	43940	*trnT-UGU-trnL-UAA*	LSC	64753	*psaJ-rpl33*	LSC	CIR, EDU, HUP, MEL, PER, TAI, UNI
24	F	30	8506	*trnG-GCC, trnG-GCC-trnR-UCU*	LSC	33970	*trnG-UCC, trnG-UCC-trnfM-CAU*	LSC	ALL

25	F	30	86465	*ycf2*	IRA	86486	*ycf2*	IRA	ALL
26	P	30	86465	*ycf2*	IRA	146921	*ycf2*	IRB	ALL
27	P	30	86486	*ycf2*	IRA	146942	*ycf2*	IRB	ALL
28	F	30	146924	*ycf2*	IRB	146945	*ycf2*	IRB	ALL
F. taipaiensis									
1	P	65	27044	*petN-psbM*	LSC	27044	*petN-psbM*	LSC	ALL
2	F	57	88615	*ycf2*	IRA	88639	*ycf2*	IRA	CIR, HUP, MEL, TAI, UNI
3	P	57	88615	*ycf2*	IRA	144432	*ycf2*	IRB	CIR, HUP, MEL, TAI, UNI
4	P	57	88639	*ycf2*	IRA	144456	*ycf2*	IRB	CIR, HUP, MEL, TAI, UNI
5	F	57	144432	*ycf2*	IRB	144456	*ycf2*	IRB	CIR, HUP, MEL, TAI, UNI
6	F	50	36104	*psaB*	LSC	38328	*psaA*	LSC	ALL
7	P	38	26820	*trnC-GCA-petN*	LSC	26820	*trnC-GCA-petN*	LSC	ALL
8	P	34	112070	*ccsA*	SSC	112070	*ccsA*	SSC	ALL
9	F	37	144452	*ycf2*	IRB	144476	*ycf2*	IRB	CIR, HUP, TAI, UNI
10	F	39	40987	*ycf3*	LSC	96018	*rps12-trnV-GAC*	IRA	CIR, EDU, HUP, KAR, PER, TAI, UNI
11	P	39	40987	*ycf3*	LSC	137071	*trnV-GAC-rps12*	IRB	CIR, EDU, HUP, KAR, PER, TAI, UNI
12	P	32	44075	*trnT-UGU-trnL-UAA*	LSC	44075	*trnT-UGU-trnL-UAA*	LSC	TAI
13	P	35	91497	*ycf2-trnL-CAA*	IRA	91497	*ycf2-trnL-CAA*	IRA	ALL
14	F	35	91497	*ycf2-trnL-CAA*	IRA	141596	*trnL-CAA-ycf2*	IRB	ALL
15	P	35	141596	*trnL-CAA-ycf2*	IRB	141596	*trnL-CAA-ycf2*	IRB	ALL
16	P	33	43973	*trnT-UGU-trnL-UAA*	LSC	43973	*trnT-UGU-trnL-UAA*	LSC	CIR, EDU, HUP, PER, TAI, UNI
17	F	38	29994	*trnT-GGU-psbD*	LSC	30007	*trnT-GGU-psbD*	LSC	CIR, TAI
18	F	37	88615	*ycf2*	IRA	88663	*ycf2*	IRA	CIR, EDU, HUP, TAI, UNI
19	P	37	88615	*ycf2*	IRA	144428	*ycf2*	IRB	CIR, EDU, HUP, TAI, UNI
20	P	37	88663	*ycf2*	IRA	144476	*ycf2*	IRB	CIR, EDU, HUP, TAI, UNI
21	F	37	144428	*ycf2*	IRB	144476	*ycf2*	IRB	CIR, EDU, HUP, TAI, UNI
22	P	34	5362	*rps16*	LSC	5362	*rps16*	LSC	CIR, EDU, TAI, UNI
23	F	31	144461	*ycf2*	IRB	144485	*ycf2*	IRB	CIR, HUP, TAI, UNI
24	P	30	112491	*ccsA-ndhD*	SSC	112534	*ccsA-ndhD*	SSC	CIR, EDU, HUP, KAR, PER, TAI, UNI
25	P	32	81104	*rpl22-rps19*	LSC	81107	*rpl22-rps19*	LSC	TAI
26	P	31	7028	*psbI-trnS-GCU, trnS-GCU*	LSC	42433	*trnS-GGA*	LSC	ALL
27	R	31	77787	*rps8-rpl14*	LSC	77787	*rps8-rpl14*	LSC	CIR, TAI, UNI
28	R	31	112616	*ccsA-ndhD, ndhD*	SSC	112616	*ccsA-ndhD, ndhD*	SSC	CIR, EDU, HUP, KAR, MEL, TAI, UNI
29	F	30	36127	*psaB*	LSC	38351	*psaA*	LSC	EDU, HUP, KAR, MEL, PER, TAI, UNI
30	P	32	33081	*psbC-trnS-UGA, trnS-UGA*	LSC	42435	*trnS-GGA, trnS-GGA-rps4*	LSC	ALL
31	F	32	88644	*ycf2*	IRA	88668	*ycf2*	IRA	CIR, HUP, TAI, UNI
32	P	32	88644	*ycf2*	IRA	144428	*ycf2*	IRB	CIR, HUP, TAI, UNI
33	P	32	88668	*ycf2*	IRA	144452	*ycf2*	IRB	CIR, HUP, TAI, UNI
34	F	31	7026	*psbI-trnS-GCU, trnS-GCU*	LSC	33082	*psbC-trnS-UGA, trnS-UGA*	LSC	ALL
35	C	31	43852	*trnT-UGU-trnL-UAA*	LSC	64496	*psaJ-rpl33*	LSC	CIR, EDU, HUP, MEL, PER, TAI, UNI
36	R	31	81159	*rpl22-rps19, rps19*	LSC	111045	*rpl32-trnL-UAG*	LSC	TAI
37	F	30	8580	*trnG-GCC, trnG-GCC-trnR-UCU*	LSC	33884	*trnG-UCC, trnG-UCC-trnfM-CAU*	LSC	ALL

38	R	30	56306	*accD-psaI*	LSC	111039	*rpl32-trnL-UAG*	SSC	MEL, TAI
39	F	30	86275	*ycf2*	IRA	86296	*ycf2*	IRA	ALL
40	P	30	86275	*ycf2*	IRA	146802	*ycf2*	IRB	ALL
41	P	30	86296	*ycf2*	IRA	146823	*ycf2*	IRB	ALL
42	F	30	146805	*ycf2*	IRB	146826	*ycf2*	IRB	ALL
F. unibracteata var. *wabuensis*									
1	P	203	107174	*ycf1*	SSC	124918	*ycf1*	SSC	UNI
2	P	65	27018	*petN-psbM*	LSC	27018	*petN-psbM*	LSC	ALL
3	F	57	88463	*ycf2*	IRA	88487	*ycf2*	IRA	CIR, HUP, MEL, TAI, UNI
4	P	57	88463	*ycf2*	IRA	143752	*ycf2*	IRB	CIR, HUP, MEL, TAI, UNI
5	P	57	88487	*ycf2*	IRA	143776	*ycf2*	IRB	CIR, HUP, MEL, TAI, UNI
6	F	57	143752	*ycf2*	IRB	143776	*ycf2*	IRB	CIR, HUP, MEL, TAI, UNI
7	F	50	36257	*psaB*	LSC	38481	*psaA*	LSC	ALL
8	P	38	26794	*trnC-GCA-petN*	LSC	26794	*trnC-GCA-petN*	LSC	ALL
9	P	34	111652	*ccsA*	SSC	111652	*ccsA*	SSC	ALL
10	F	37	143772	*ycf2*	IRB	143796	*ycf2*	IRB	CIR, HUP, TAI, UNI
11	F	39	41146	*ycf3*	LSC	95606	*rps12-trnV-GAC*	IRA	CIR, EDU, HUP, KAR, PER, TAI, UNI
12	P	39	41146	*ycf3*	LSC	136651	*trnV-GAC-rps12*	IRB	CIR, EDU, HUP, KAR, PER, TAI, UNI
13	P	35	91085	*ycf2-trnL-CAA*	IRA	91085	*ycf2-trnL-CAA*	IRA	ALL
14	F	35	91085	*ycf2-trnL-CAA*	IRA	141176	*trnL-CAA-ycf2*	IRB	ALL
15	P	35	141176	*trnL-CAA-ycf2*	IRB	141176	*trnL-CAA-ycf2*	IRB	ALL
16	P	33	44111	*trnT-UGU-trnL-UAA*	LSC	44111	*trnT-UGU-trnL-UAA*	LSC	CIR, EDU, HUP, PER, TAI, UNI
17	R	35	43995	*trnT-UGU-trnL-UAA*	LSC	43995	*trnT-UGU-trnL-UAA*	LSC	UNI
18	F	37	88463	*ycf2*	IRA	88511	*ycf2*	IRA	CIR, EDU, HUP, TAI, UNI
19	P	37	88463	*ycf2*	IRA	143748	*ycf2*	IRB	CIR, EDU, HUP, TAI, UNI
20	P	37	88511	*ycf2*	IRA	143796	*ycf2*	IRB	CIR, EDU, HUP, TAI, UNI
21	F	37	143748	*ycf2*	IRB	143796	*ycf2*	IRB	CIR, EDU, HUP, TAI, UNI
22	P	34	5381	*rps16*	LSC	5381	*rps16*	LSC	CIR, EDU, TAI, UNI
23	F	31	143781	*ycf2*	IRB	143805	*ycf2*	IRB	CIR, HUP, TAI, UNI
24	F	36	30134	*trnT-GGU-psbD*	LSC	30156	*trnT-GGU-psbD*	LSC	UNI
25	P	30	112073	*ccsA-ndhD*	SSC	112116	*ccsA-ndhD*	SSC	CIR, EDU, HUP, KAR, PER, TAI, UNI
26	P	31	7059	*psbI-trnS-GCU, trnS-GCU*	LSC	42594	*trnS-GGA*	LSC	ALL
27	R	31	77639	*rps8-rpl14*	LSC	77639	*rps8-rpl14*	LSC	CIR, TAI, UNI
28	R	31	112198	*ccsA-ndhD, ndhD*	SSC	112198	*ccsA-ndhD, ndhD*	SSC	CIR, EDU, HUP, KAR, MEL, TAI, UNI
29	F	30	36280	*psaB*	LSC	38504	*psaA*	LSC	EDU, HUP, KAR, MEL, PER, TAI, UNI
30	P	32	33234	*psbC-trnS-UGA, trnS-UGA*	LSC	42596	*trnS-GGA, trnS-GGA-rps4*	LSC	ALL
31	F	32	88492	*ycf2*	IRA	88516	*ycf2*	IRA	CIR, HUP, TAI, UNI
32	P	32	88492	*ycf2*	IRA	143748	*ycf2*	IRB	CIR, HUP, TAI, UNI
33	P	32	88516	*ycf2*	IRA	143772	*ycf2*	IRB	CIR, HUP, TAI, UNI
34	F	31	7057	*psbI-trnS-GCU, trnS-GCU*	LSC	33235	*psbC-trnS-UGA, trnS-UGA*	LSC	ALL
35	C	31	43969	*trnT-UGU-trnL-UAA*	LSC	64369	*psaJ-rpl33*	LSC	CIR, EDU, HUP, MEL, PER, TAI, UNI
36	C	30	1663	*trnK-UUU*	LSC	81000	*rpl22-rps19*	LSC	UNI

37	F	30	7596	*trnS-GCU-trnG-GCC*	LSC	7610	*trnS-GCU-trnG-GCC*	LSC	MEL, UNI
38	F	30	8544	*trnG-GCC, trnG-GCC-trnR-UCU*	LSC	34037	*trnG-UCC, trnG-UCC-trnfM-CAU*	LSC	ALL
39	R	30	45396	*trnL-UAA-trnF-GAA*	LSC	46020	*trnF-GAA-ndhJ*	LSC	UNI
40	R	30	81006	*rpl22-rps19, rps19*	LSC	110629	*rpl32-trnL-UAG*	SSC	UNI
41	F	30	86123	*ycf2*	IRA	86144	*ycf2*	IRA	ALL
42	P	30	86123	*ycf2*	IRA	146122	*ycf2*	IRB	ALL
43	P	30	86144	*ycf2*	IRA	146143	*ycf2*	IRB	ALL
44	F	30	146125	*ycf2*	IRB	146146	*ycf2*	IRB	ALL
